# Evolution and Vulnerability of the Global Ready-to-Eat Aquatic Products Trade Network: A Complex Network Analysis

**DOI:** 10.3390/foods15101648

**Published:** 2026-05-09

**Authors:** Xiaonan Fan, Shenghui Sun, Lixin Zheng, Yang Liu, Weihua Yang, Dongmei Li

**Affiliations:** 1 School of Management, Dalian Polytechnic University, Dalian 116034, China; fanxiaonan@dlpu.edu.cn (X.F.); 18342276835@163.com (S.S.); zhenglixinxxx@163.com (L.Z.); yangwh@dlpu.edu.cn (W.Y.); 2 National Engineering Research Center of Seafood, School of Food Science and Technology, Dalian Polytechnic University, Dalian 116034, China; 426417898@qq.com; 3 State Key Laboratory of Marine Food Processing & Safety Control, Dalian Polytechnic University, Dalian 116034, China

**Keywords:** global ready-to-eat Aquatic Products, network evolution, vulnerability, complex networks, trade networks

## Abstract

Against the background of increasing complexity in global aquatic food supply chains and rising trade risks, the structural stability and vulnerability of ready-to-eat Aquatic Products trade networks have become increasingly prominent. This study applies complex network analysis to examine the evolution and vulnerability of the overall trade network and its subnetworks. The results show that (1) the overall trade network exhibits significant small-world characteristics; (2) the core countries in the network are China, the United States, Thailand, France, and Spain, which play dominant roles; (3) the community structure of the overall network is clearer, with stronger intra-community trade links; (4) all subnetworks also exhibit small-world properties, and different categories of ready-to-eat Aquatic Products have different core countries; (5) vulnerability analysis shows that the network is sensitive to targeted attacks, non-fish subnetworks are more vulnerable, while fish subnetworks are more stable. These findings provide practical guidance for procurement decisions, supply chain configuration, and risk management in the ready-to-eat Aquatic Products industry.

## 1. Introduction

Global Aquatic Products trade has shown increasing uncertainty in recent years. The total trade volume decreased from 28.88 million tons in 2020 to 28.51 million tons in 2023. This change is not only driven by supply–demand adjustments, but also closely related to structural features of the trade network. These include node dependence, link fragility, and cross-regional transmission risks. Disruptions in network configuration, connection patterns, or operational mechanisms can directly affect trade volume and supply stability. Ready-to-eat Aquatic Products are a high-value segment of aquatic products. They involve complex processing, high technical requirements, strong convenience, and heavy reliance on cold-chain logistics [1,2]. They also require strict time control and intensive cross-border coordination. Therefore, they are more vulnerable to shocks such as geopolitical conflicts, logistics disruptions, public health events, and market fluctuations. Understanding the evolution and vulnerability of the global ready-to-eat Aquatic Products trade network is essential for identifying trade risks and improving industry resilience.

From a theoretical perspective, the risks faced by the global ready-to-eat Aquatic Products trade network cannot be simply understood as ordinary trade fluctuations, nor as the simple sum of several isolated shocks. Instead, they are better interpreted within the framework of polycrisis. Polycrisis theory emphasizes that crises in different systems interact through causal links, feedback mechanisms, and cross-system transmission. These interactions can reinforce one another and generate cascading effects across global, regional, and local scales [3,4]. In the trade of ready-to-eat Aquatic Products, factors such as climate pressure, fluctuations in processing costs, disruptions in cold-chain logistics and port operations, trade policy adjustments, and changes in market demand do not exist independently. Rather, they affect one another through production, processing, logistics, governance, and consumption, and jointly drive changes in network structure while intensifying risk diffusion [5,6]. Therefore, the dynamic changes in the global ready-to-eat Aquatic Products trade network should be viewed as a structural expression of systemic vulnerability under a polycrisis context. Specifically, this vulnerability is reflected not only in the dependence on a few core countries and key trade routes, but also in the possibility that risks such as pandemics, geopolitical fragmentation, logistics disruptions, and climate pressure may reinforce each other and spread across regions along trade links.

Complex network analysis is an important tool for analyzing the evolution and vulnerability of the ready-to-eat Aquatic Products trade network in the context of multiple crises. Although this method cannot directly identify all causal mechanisms among the elements of multiple crises, it can effectively reveal the connectivity, concentration, and potential cascading risks of the trade network under complex shocks. This advantage has also been fully demonstrated in existing studies on food trade networks. Relevant studies have shown that the topological structure, distribution of core nodes, and community organization of food trade networks affect not only food availability and food safety, but also the resilience of networks under random and targeted shocks [7,8,9,10,11,12]. Building on this, research has gradually expanded to specific agricultural products, such as soybeans, wheat, rice, and maize, as well as the overall agricultural trade network, revealing significant differences across food trade systems in core countries, network concentration, community reorganization, and risk diffusion mechanisms [13,14,15,16,17,18,19,20,21,22,23]. In the field of aquatic products, existing studies have mainly focused on the overall Aquatic Products trade network, regional trade restructuring, and the trade characteristics of fish products, and have preliminarily revealed the dynamic changes in the Aquatic Products trade system under the constraints of spatial organization, policy drivers, and sustainability [24,25,26,27,28,29]. These studies have laid an important foundation for understanding Aquatic Products trade networks. However, they have mainly focused on overall aquatic products or single fish products. Systematic discussion is still lacking for ready-to-eat Aquatic Products, a segment with stronger processing attributes, a higher degree of supply chain coordination, and more complex risk transmission.

Based on the existing literature, this paper argues that current research has at least two limitations. First, existing studies mainly focus on the trade networks of general food, grain, or overall aquatic products. They lack dedicated analysis of ready-to-eat Aquatic Products as a high-value-added segment, especially comparative analysis across different categories of ready-to-eat Aquatic Products. Second, although factors such as the pandemic, geopolitical conflicts, logistics disruptions, market concentration, and environmental uncertainty are often used to explain trade fluctuations, existing studies mostly treat them as an external background. They have not systematically examined, from the perspective of complex networks in the context of multiple crises, how these factors interact through network structures and intensify systemic vulnerability. These gaps limit our understanding of the evolutionary mechanisms and risk diffusion processes of the global ready-to-eat Aquatic Products trade system.

To address the above research gaps, this paper aims to analyze the structural evolution and vulnerability characteristics of the global ready-to-eat Aquatic Products trade network from the perspective of complex networks in the context of multiple crises. Specifically, this paper focuses on three questions: What structural evolution characteristics did the global ready-to-eat Aquatic Products trade network show during 2011–2023? What differences existed among the sub-trade networks of different categories of ready-to-eat Aquatic Products in terms of topological properties and the distribution of core countries? How did the overall trade network and different sub-trade networks perform in terms of vulnerability under random attack and targeted attack scenarios? To answer these questions, this paper constructs the overall trade network and sub-trade networks for ready-to-eat fish products, shrimp products, crab products, shellfish products, cephalopod products, and other ready-to-eat Aquatic Products. Based on global bilateral trade data from 2011 to 2023. Complex network analysis is used to describe network evolution from the dimensions of network density, average path length, clustering coefficient, centrality indicators, and community structure. Network vulnerability is then evaluated through simulations of random attacks and targeted attacks.

The remainder of this paper is organized as follows. Section 2 introduces the data sources, classification procedures, and complex network analysis methods. Section 3 presents the evolutionary characteristics and vulnerability analysis results of the global overall trade network for ready-to-eat Aquatic Products and its sub-trade networks. Section 4 discusses the main findings and explains changes in network structure and systemic risks from the perspective of multiple crises. Section 5 concludes the paper and summarizes its academic contributions, research limitations, and future prospects.

## 2. Methods and Data

### 2.1. Methodological Framework

This study used complex network analysis to examine the structural evolution of the global RTEAP-TN from 2011 to 2023. In this analytical framework, countries or regions engaged in ready-to-eat Aquatic Products trade were treated as nodes in the trade network, and trade relationships between countries or regions were represented as edges. Edge direction followed trade flows, from exporter to importer, and trade volume was used as the edge weight. On this basis, a directed and weighted global RTEAP-TN was constructed. The trade network is represented by two sets: the node set *V*(*G*) and the edge set *E*(*G*), where *V* = {*v_i_*: i = 1, 2, …, *n*}, and n is the number of nodes in the network, and *E* = {*e_k_*: *k* = 1, 2, …, *m*}, and *m* is the number of edges in the network.

Based on the constructed directed and weighted network, this study calculated degree centrality, closeness centrality, betweenness centrality, eigenvector centrality, PageRank centrality, average clustering coefficient, average path length, and network density. All calculations were performed using the built-in statistical modules of Gephi (version 0.10.1). Specifically, the processed edge list was imported into Gephi to construct the network, and its built-in modularity optimization algorithm was used to identify and visualize community structure, with edge weights enabled and the resolution parameter set to 1.

**Figure 1:**
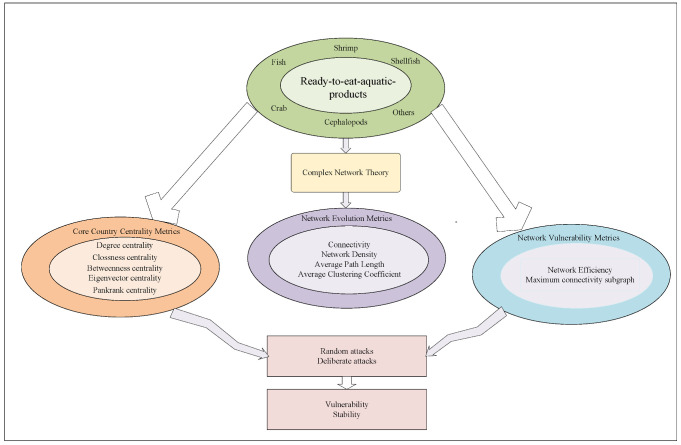
Research Methodology and Framework of This Paper.

On this basis, this study conducted vulnerability simulations using the overall trade network in 2023. Targeted attacks were simulated using an adaptive strategy. After each node removal, node importance in the remaining network was recalculated to determine the target for the next round of removal. Random attacks were simulated through 100 independent runs, and the results were reported as the mean and the 95% confidence interval. Network vulnerability was evaluated based on changes in network efficiency and the share of the largest connected component. The evaluation framework is shown in Figure 1. Vulnerability simulations, including random attacks and targeted attacks, were conducted using R software, version 4.4.3.

### 2.2. Metrics

The complexity of the global RTEAP-TN exhibits significant multidimensional characteristics, making it difficult for a single evaluation indicator to provide a comprehensive and accurate characterization. As a structural representation of the global food supply chain in the international trade segment, this trade network not only directly reflects the patterns of commodity flows among countries and regions, but also clearly reveals actor linkages, hierarchical divisions, and interdependencies within the global supply chain system. Existing studies on supply chain complexity have demonstrated that supply chain complexity can be quantitatively characterized through the core structural features of supply networks, including network size, relational connections, structural hierarchy, and network dependence. This provides a fundamental theoretical basis for constructing an evaluation indicator system for trade network complexity from the perspective of complex network theory [30,31]. Based on this, this study selects five core dimensions—network scale, trade connectivity, node importance, community structure, and system vulnerability—and systematically analyzes the structural complexity of the global RTEAP-TN using multidimensional structural indicators.

(1) Network size

This dimension uses the most basic structural indicators to define the overall scope of participation and the volume of trade linkages in the global RTEAP-TN. It provides the basic foundation for network analysis. The number of nodes refers to the total number of countries/regions participating in ready-to-eat Aquatic Products trade in the network. It is a core indicator for measuring the size of participating actors. The number of edges refers to the total number of effective trade links that actually exist among countries/regions in the network. It reflects the overall scale of actual trade exchanges in the trade network.

(2) Degree of Trade Integration

The degree of Trade Integration is represented by the average degree, network density, average path length, and average clustering coefficient.

The average degree, denoted by *β*, represents the average number of edges connected to each node. The formula is as follows:

(1) β=m/n

where *m* is the total number of edges in the network, and *n* is the total number of nodes in the network.

Although both the average degree and network density describe the degree of network connectivity, they differ in their perspectives. The average degree focuses on the actual number of connections of each node, whereas network density emphasizes the ratio of the actual number of edges to the theoretical maximum number of possible edges, thereby reflecting the saturation level of the network. The relationship between the two is as follows:

(2) β=D×(n−1)

Network density, denoted by *D*, reflects the closeness of connections among countries or regions in the international trade network. A higher density indicates closer connections. The formula is as follows:

(3) $$D=\frac{M}{N(N-1)}\;$$

where *M* is the actual number of edges in the network.

The average clustering coefficient, denoted by *C*, reflects the degree of clustering among countries in the RTEAP-TN. A higher clustering coefficient indicates a higher level of local aggregation around a node [32]. The formula is as follows:

(4) $$C=\frac{1}{s_{i}(k_{i}-1)}\sum_{j,h}\left(\frac{w_{ij}+w_{ih}}{2}\right)a_{ij}a_{ih}a_{jh}$$

where *s_i_* denotes the strength of node *i*, that is, the sum of the weights of all edges connected to it; *ki* denotes the degree of node *i*, that is, the number of its neighboring nodes; *w_ij_*, *w_ih_*, and *w_jh_* denote the weights of the edges between nodes *i* and *j*, *i* and *h*, and *j* and *h*, respectively; and *a_ij_*, *a_ih_* and *a_jh_* denote the elements of the adjacency matrix. If there is an edge between nodes *i* and *j*, then *a_ij_* = 1; otherwise, *a_ij_* = 0.

The average path length, denoted by *L*, is the average of the shortest path lengths between all reachable pairs of nodes in the network. It is mainly used to measure the overall efficiency of global connectivity. A smaller value indicates a higher overall connectivity efficiency of the network. The formula is as follows:

(5) $$L=\frac{2}{N(N-1)}\sum_{i\geq j}^{N}d_{ij}$$

where *dij* represents the shortest path length from node *i* to node *j*, and *N*(*N* − 1) represents the number of all possible directed node pairs in the network.

(3) Node importance

The evaluation of node importance includes degree centrality, closeness centrality, betweenness centrality, eigenvector centrality, and PageRank centrality.

Degree centrality, denoted by *CD*(*i*), is the most direct indicator in complex network analysis. It is used to measure the proportion of a node’s direct connections to the maximum possible number of connections in the network [33]. For nodes in a directed and weighted network, degree centrality can be divided into in-degree centrality, *C_D_*_,in_(*ni*), and out-degree centrality, *C_D_*_,out_(*ni*). The formulas are as follows:

(6) $$C_{D,\mathrm{in}}(n_{i})=\frac{1}{n-1}\sum_{j=1}^{n}r_{ji,\mathrm{in}}$$

(7) $$C_{D,\mathrm{out}}(n_{i})=\frac{1}{n-1}\sum_{j=1}^{n}r_{ij,\mathrm{out}}$$

where *r_ij_*_,in_ denotes the strength of the incoming relationship from node *j* to node *i*, which in this study is measured by trade volume; *r_ij_*_,out_ denotes the strength of the outgoing relationship from node *i* to node *j*, which in this study is also measured by trade volume; *n* denotes the total number of nodes; and *n* − 1 denotes the maximum possible number of connections of a node.

Closeness centrality, denoted by *C_C_*(*i*) is an indicator that evaluates the efficiency of a node’s global reachability by measuring the sum of the shortest path lengths from that node to all other nodes in the network. The shorter the average shortest path of a node, the higher its closeness centrality and the faster the transmission of information or resources [34]. The formula is as follows:

(8) $$C_{c}\left(i\right)=\frac{n-1}{\sum_{j\neq i\in N}d_{ij}}$$

where *d_ij_* denotes the shortest distance between node *i* and node *j*, and *N* denotes the number of countries.

Betweenness centrality, denoted by *C_B_*(*x*), measures the extent to which a single node controls the shortest paths between pairs of nodes in the network [35]. The formula is as follows:

(9) $$C_{B}(x)=\frac{\left[2\sum_{i\neq j\neq x}\frac{\sigma_{ij}(x)}{\sigma_{ij}}\right]}{\left(N-1)(N-2\right)}$$

where *σ_ij_*(x) denotes the number of shortest paths from node *i* to node *j* that pass through node *x*; (*N* − 1)(*N* − 2) is a normalization factor used to ensure that the centrality value is not affected by network size; and *σ_ij_*(*x*)/*σ_ij_* denotes the proportion of shortest paths between nodes *i* and *j* that pass through node *x*.

Eigenvector centrality, denoted by *C_E_*(*i*), is a centrality indicator used to measure the global influence of a node. Its core idea is that the importance of a node depends not only on the number of its connections, but also on the quality of those connections. In other words, a node must have both a relatively large number of connections and highly important connected nodes in order to have high eigenvector centrality [36]. The formula is as follows:

(10) $$C_{E}(i)=x_{i}=\frac{1}{\lambda }\sum_{j=1}^{n}A_{ij}x_{j}$$

where *x_j_* denotes the eigenvector centrality value of node *j*; *A_ij_* denotes the element in row *i* and column *j* of the network adjacency matrix; and *λ* is a constant factor.

PageRank centrality, denoted by *PR*(*i*), refers to the probability that a country or region has a trade link with another country or region and further connects with other countries or regions through that link. The higher the PageRank centrality of a country or region, the more important its hierarchical position and the stronger its leading role in the global trade network [37]. The formula is as follows:

(11) $$PR(i)=\frac{1-d}{\left|N\right|}+d\sum_{j\in M(i)}\frac{PR(j)}{L(j)}$$

where *p* is the damping factor, which ensures that a jump can be made from a node with no links to another random node, and it is usually set to 0.85; *M*(*i*) denotes the set of nodes that link to node *i*; *L*(*j*) denotes the total number of outgoing links from node *j*; and ∣*N*∣ denotes the total number of nodes in the network.

(4) Community analysis

Community analysis aims to identify the group with which a country is most closely connected in the trade network, as well as its position within that community. In the specific calculation process, a modularity optimization algorithm is used [38]. The formula is as follows:

(12) $$Q_{w}=\frac{1}{2W}\sum_{C\in P}\sum_{i,j\in C}\left[w_{ij}-\frac{s_{i}s_{j}}{2W}\right]$$

where *W* is the sum of the weights of all edges in the network; *w_ij_* is the weight of the edge between node *i* and node *j*; *s_i_* is the sum of the weights of all edges connected to node *i*; and *s_j_* is the sum of the weights of all edges connected to node *j*.

(5) Vulnerability indicators

Network efficiency, denoted by *E*, is the average of the reciprocals of the shortest path lengths between all pairs of nodes in the network. As a key parameter for measuring overall network performance, it is closely related to network stability [39]. The formula is as follows:

(13) $$E=\frac{2}{N(N-1)}\sum_{\begin{array}{c} i,j\in N\\ i\neq j \end{array}}\frac{1}{d_{ij}}$$

The proportion of the largest connected component, denoted by *S*, represents the share of the largest connected component in the total number of nodes in the network. Depending on the graph type, this may refer to the largest connected component or the largest weakly connected component. It reflects the strength of network connectivity after node removal. The formula is as follows:

(14) $$S=\frac{N_{\mathrm{largest}}}{N}$$

where *N*_largest_ denotes the number of nodes contained in the largest connected component. In this study, the weak connectivity definition is adopted to reflect the overall connectivity of the network when edge directions are ignored.

(6) Classical network structure

The small-world network was first proposed by Watts and Strogatz in 1998 [40]. It refers to a network state between regular and random networks, characterized by strong local clustering and a small number of long-range connections. Traditionally, small-world properties are mainly described by the clustering coefficient *C* and the characteristic path length *L*. Considering that a descriptive judgment based only on a high clustering coefficient and a short path length is insufficient to rigorously support a small-world conclusion, this study further introduces a comparison method based on random null models to test the small-world property of the RTEAP-TN. Specifically, this study calculates the clustering coefficient of the observed network *C*_obs_ and the characteristic path length of the observed network *L*_obs_. It then constructs a set of random networks that preserve network size and degree sequence in order to obtain the average clustering coefficient of the random networks *C*_rand_ and the average characteristic path length of the random networks *L*_rand_. Based on these measures, the following definitions are given:

(15) $$\lambda =\frac{L_{\mathrm{obs}}}{\langle L_{\mathrm{rand}}\rangle }$$

(16) $$\gamma =\frac{C_{\mathrm{obs}}}{\langle C_{\mathrm{rand}}\rangle }$$

(17) $$\sigma =\frac{\gamma }{\lambda }$$

where *σ* is the small-world coefficient. A value of *σ* > 1 indicates that, compared with the random network, the observed network has stronger local clustering while maintaining a similar path length. Therefore, the network as a whole can be regarded as having small-world properties.

The scale-free network was formally proposed by Albert-László Barabási and his student Réka Albert in 1999. It implies that a small number of countries account for most trade relationships in the network. In complex networks, a network is considered scale-free if its degree distribution follows a power-law distribution [41].

(18) $$p(k)\sim k^{-\gamma }$$

where *p*(*k*) is the degree distribution, *k* is the degree of a node, and *γ* is the power-law exponent.

The scale-free property is tested by directly fitting the degree distribution using maximum likelihood estimation, which automatically estimates the power-law exponent *γ* and the lower bound of the tail, *x*_min_. After fitting, the Kolmogorov–Smirnov test is used to assess goodness of fit. The Vuong test is then used to compare the power-law distribution with the lognormal and exponential distributions to ensure the plausibility of the power-law fit.

### 2.3. Data Sources and Processing

The ready-to-eat Aquatic Products trade data used in this study were obtained from the United Nations Comtrade Database (UN Comtrade). It should be noted that the focus of this study is not primary aquatic products in the general sense, but ready-to-eat Aquatic Products with a certain degree of processing that can be consumed directly or after simple heating. Therefore, in terms of product classification, this study did not directly adopt the existing classification standards of the Codex Alimentarius Commission (CAC) or the European Union for raw aquatic products. Instead, it refined the classification of the study objects by drawing on their basic logic of fish-crustacean-mollusc categories, while also considering the semi-processed and convenience-oriented characteristics of ready-to-eat Aquatic Products, as well as the classification approaches used in previous studies on processed Aquatic Products [42,43]. Specifically, this study divided ready-to-eat Aquatic Products into six categories ready-to-eat fish products, shrimp products, crab products, shellfish products, cephalopod products, and other ready-to-eat Aquatic Products (Supplementary S1). In terms of data processing, this study first established an HS six-digit product code mapping system and extracted global bilateral trade data from 2011 to 2023. It then removed trade records with annual trade volumes below 500 kg to reduce the interference of occasional small-volume transactions with the network structure.

## 3. Results and Analysis

### 3.1. Evolutionary Analysis of the RTEAP-TN Overall Trade Network

#### 3.1.1. Development Overview

**Figure 2:**
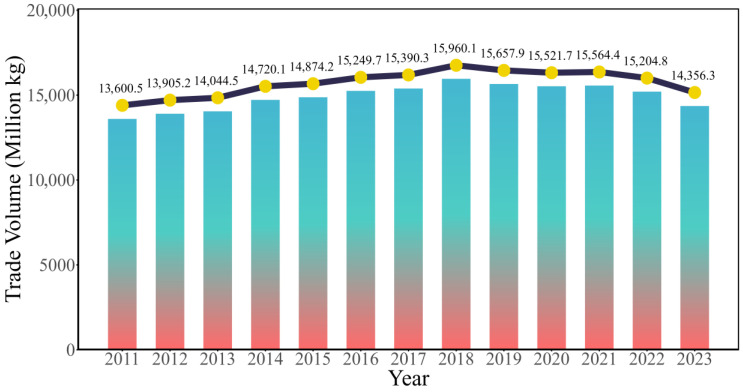
Trends in the Trade Volume of Ready-to-eat Aquatic Products.

To identify the overall evolutionary trend of the global ready-to-eat Aquatic Products trade network, this study first examines changes in trade volume and its spatial organizational characteristics from 2011 to 2023. The global trade volume of ready-to-eat Aquatic Products generally follows a trajectory of “growth first, then decline” (Figure 2). From 2011 to 2018, trade volume increased from 13.6005 million tons to 15.9601 million tons, showing relatively rapid overall growth. After 2018, trade volume entered a stage of fluctuating decline. Although a certain rebound appeared in 2021, the overall level still did not return to its peak. This reflects the combined effects of multiple headwinds, including protectionist policies, geopolitical tensions, and logistics disruptions caused by the pandemic.

**Figure 3:**
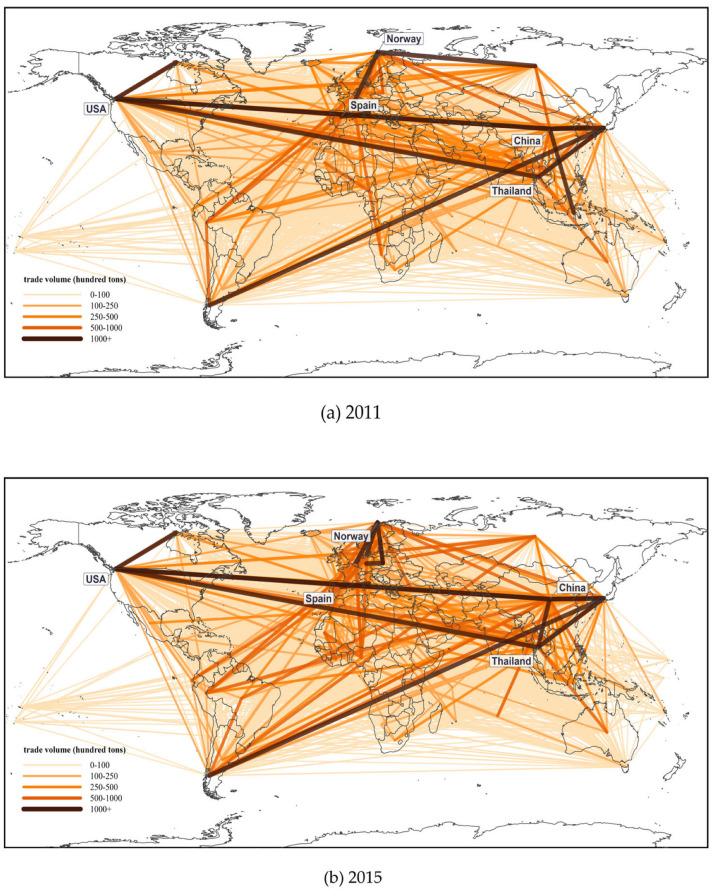

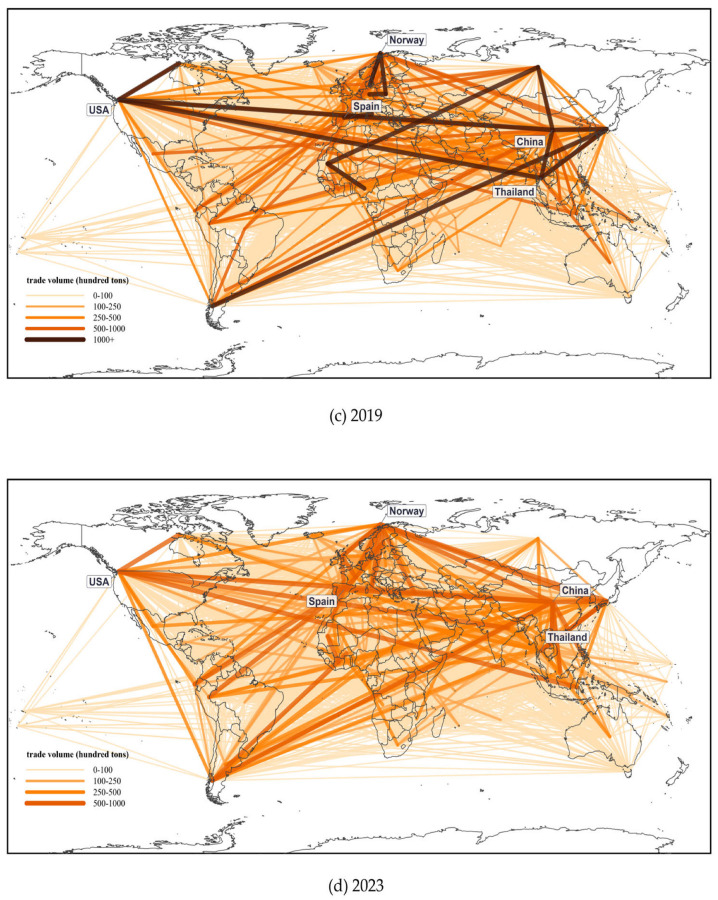
Spatiotemporal Evolution of the RTEAP-TN. The shade of the lines indicates the volume of trade. The five highlighted nodes represent the five countries with the largest trade volume in the corresponding year.

From a spatial perspective, Figure 3 reveals that the global RTEAP-TN has clear regional agglomeration characteristics. First, the Asia-Pacific region and Europe constitute the two most active core blocs in the global ready-to-eat Aquatic Products trade, and trade linkages within each region are relatively close. Second, trade relations between neighboring countries remain relatively active, such as those among East Asian countries and among North American countries. This indicates that geographic proximity, logistics convenience, and similarity in consumption structure remain important factors affecting trade linkages. Third, cross-regional trade has not spread evenly. Instead, it is mainly concentrated among a few core countries, especially along major trade corridors linking Asia, Europe, and North America. Overall, the expansion of the global ready-to-eat Aquatic Products trade network has not led to an even dispersion of trade relations. Rather, it has further strengthened the characteristics of regional agglomeration and dependence on key cross-regional corridors.

#### 3.1.2. Structural Characteristics

Building on the overall development pattern, this study further examined changes in the network structure of the RTEAP-TN during the study period. From 2011 to 2023, the number of network nodes remained generally stable, whereas the number of edges increased from 5863 to 6026, the average degree rose from 30.70 to 31.22, network density increased slightly from 0.1616 to 0.1626, the average clustering coefficient improved from 0.599 to 0.604, and the average path length shortened from 1.993 to 1.975 (Table 1). Overall, the RTEAP-TN showed an evolutionary pattern of stronger linkages, deeper local clustering, and slightly improved global accessibility during the study period.

It should be noted that this strengthening trend experienced a temporary fluctuation in 2020. In that year, the average degree and network density declined, while the average path length increased, indicating that some trade linkages were disrupted and that the overall connectivity efficiency of the network weakened. This suggests that although the RTEAP-TN generally continued to strengthen, it still exhibited considerable structural sensitivity under major external shocks.

From a topological perspective, the small-world index of the RTEAP-TN was greater than 1 in all years, indicating that the network had both strong local clustering and high overall connectivity efficiency. This supports the conclusion that the RTEAP-TN had small-world characteristics. Further degree distribution tests showed that the node degree distribution displayed a clear heavy-tail pattern, and the upper tail exhibited some power-law behavior. However, a strict scale-free interpretation should still be treated with caution. Even so, this finding implies that the global ready-to-eat Aquatic Products trade was highly concentrated in a few core countries, whereas most countries maintained trade relations with only a limited number of partners (Supplementary S2).

This “small-world + hub concentration” structure had dual effects. On the one hand, it facilitated the rapid global flow of trade information, products, and resources. On the other hand, it also meant that the network depended heavily on a few core nodes. Once key nodes were disrupted, risks could spread rapidly along efficient connection paths, thereby amplifying systemic vulnerability.

These results were generally consistent with previous studies on global Aquatic Products trade networks and agricultural trade networks. Gephart and Pace (2015) [44] pointed out in their study of the global Aquatic Products trade network that Aquatic Products trade networks show strong connectivity and clear dependence on core nodes. Yu and Ma (2020) and Wang and Dai (2021) [8,24] also found that international Aquatic Products and food trade networks generally exhibit strong local clustering, short average path lengths, and pronounced hub-concentrated structures. The present results further indicate that these structural characteristics were also evident in the more processing-intensive segment of ready-to-eat Aquatic Products.

#### 3.1.3. Analysis of Core Countries

**Figure 4:**
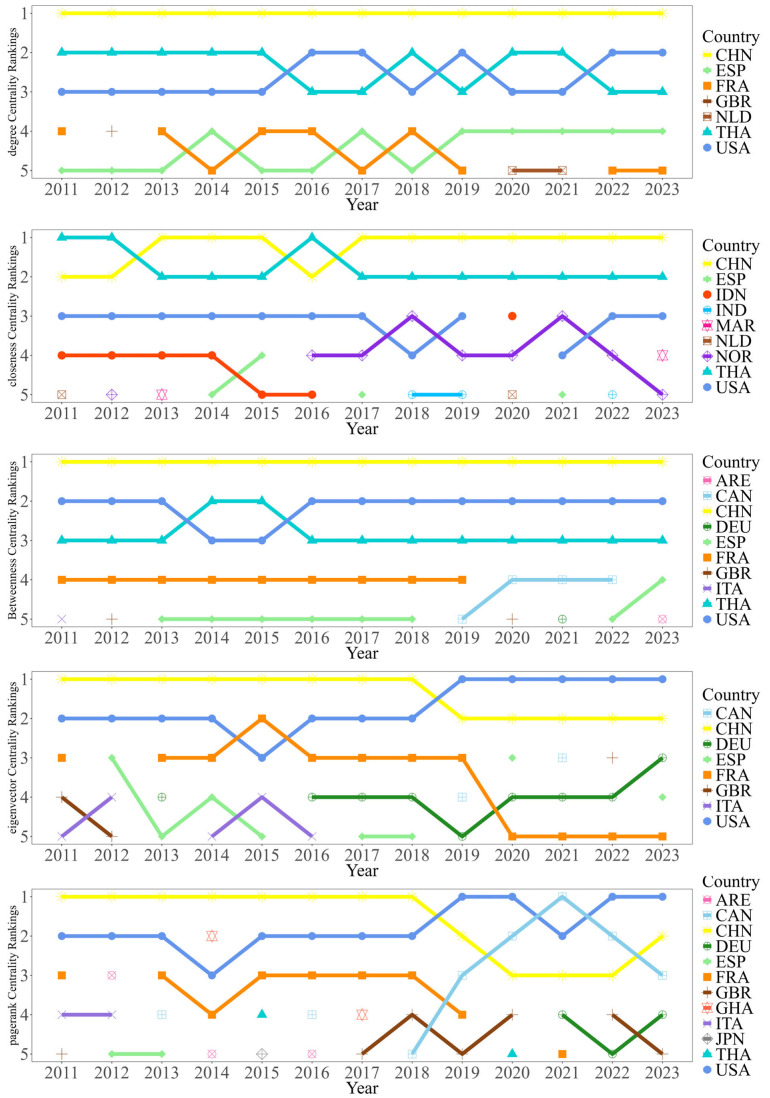
Ranking of major trading entities in the RTEAP-TN based on centrality metrics.

To further identify the key countries that sustain the operation of the overall trade network, this study compared the performance of each country across multiple centrality indicators. China, the United States, Thailand, France, and Spain consistently rank among the top countries in several centrality measures and together form the stable core group of the RTEAP-TN (Figure 4).

These core countries do not perform exactly the same functions in the network. China performs strongly in degree centrality, closeness centrality, and betweenness centrality. This indicates that it has extensive trade connections, strong network accessibility, and a significant bridging and coordinating role. The United States also occupies a core position in the network, reflecting its role as an important final consumer market and a key trade node. Thailand stands out in degree centrality and betweenness centrality, showing that it plays an important role in the global processing and entrepot trade of ready-to-eat Aquatic Products. France and Spain are more important as key nodes in regional trade integration and cross-regional linkages. Overall, the RTEAP-TN is not dominated by a single country. Instead, it is jointly supported by a group of core countries with clearly differentiated functions.

#### 3.1.4. Community Evolution Analysis

**Figure 5:**
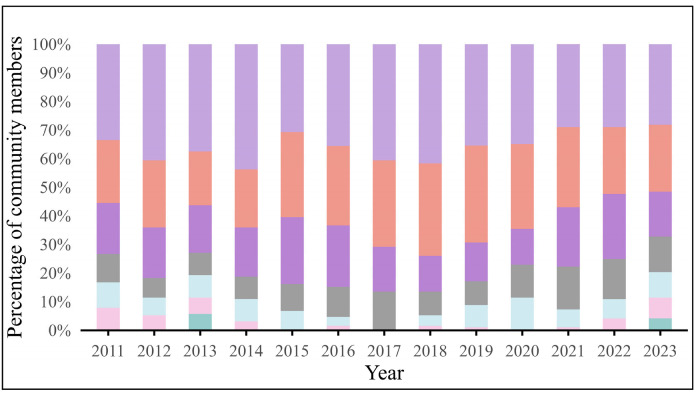
Community member ratio.

The community formation of the global ready-to-eat Aquatic Products trade network is increasingly moving away from geographic proximity and instead reflecting geopolitical alignments. During this period, the modularity index of the RTEAP-TN fluctuates slightly between 0.3 and 0.4, indicating that the community structure shows relatively good stability (Table 2). The number of communities remains basically between 4 and 7, suggesting a relatively stable pattern. The size distribution of different communities is uneven across years. Some communities include more members, whereas others have relatively few members (Figure 5).

**Figure 6:**
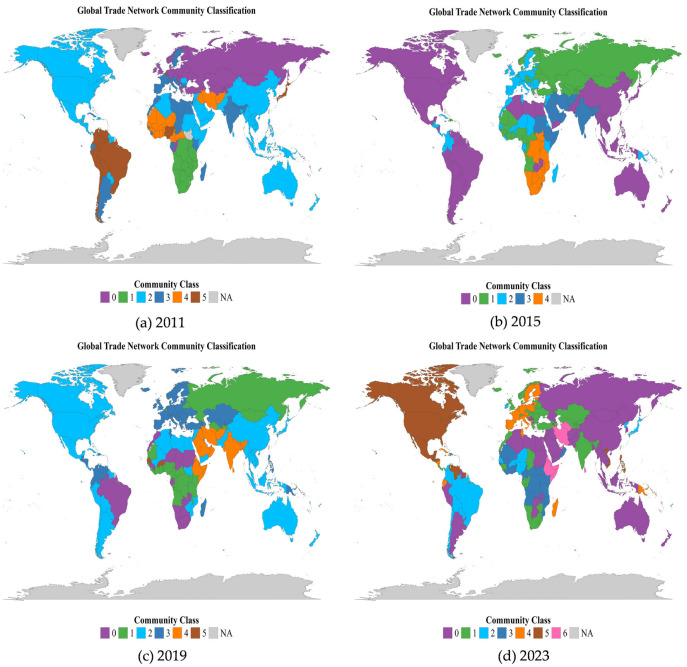
Community Evolution of RTEAP-TN.

The community evolution of the global ready-to-eat Aquatic Products trade network is primarily driven by geopolitics and great-power competition. The RTEAP-TN exhibits a coexistence of regional agglomeration and cross-regional interconnection (Figure 6). The European community shows relatively clear boundaries and a high degree of regionalization, indicating strong internal cohesion. In contrast, communities involving countries in the Asia-Pacific, the Middle East, and Africa display more fluid and less clearly defined boundaries.

To capture the dynamics of community evolution, this study adopts a four-year time window. A one-year interval is often insufficient to reveal structural trends, whereas a four-year period allows clearer identification of evolutionary trajectories without obscuring critical turning points.

In 2011, the RTEAP-TN consisted of six communities, among which two were dominant in size. The largest community, centered on China and the United States, included 64 economies and was primarily composed of North American and Asian countries. The second-largest community, centered on India, the European Union, and Russia, comprised 42 economies, with members mainly distributed across Asia and Europe.

By 2015, the community structure had undergone significant reorganization. The largest community was further consolidated. With the advancement of the Trans-Pacific Partnership and the upgrading negotiations of the China–Chile Free Trade Agreement, South American countries were increasingly integrated into the trade system centered on China and the United States, strengthening community cohesion and reinforcing its central position. In contrast, the second-largest community experienced fragmentation. The India–EU relationship reached a turning point as free trade negotiations stalled. India shifted its trade focus toward the Middle East to compensate for declining engagement with the European market, weakening the foundation of the original India–EU community. Meanwhile, the European Union, supported by technological barriers, formed a more internally cohesive and clearly bounded community. In addition, following the Ukraine crisis in 2014, Russia withdrew from the EU-led community, accelerating its restructuring.

In 2019, the RTEAP-TN exhibited a parallel trend of multipolarization and regionalization. The China–US-centered community maintained its scale advantage, while several medium-sized economies began to diversify trade partnerships to mitigate external risks. For instance, Brazil gradually aligned with a trade system centered on African economies such as Morocco and South Africa. The European Union strengthened its internal integration by incorporating countries such as Kazakhstan, thereby enhancing structural stability. Under continued EU sanctions, Russia actively expanded trade relations with African countries, including Ghana and Angola, through platforms such as the first Russia–Africa Summit. This reflects a strategic shift toward regional cooperation outside the mainstream trade system.

By 2023, under rising protectionism, persistent geopolitical conflicts, sluggish global economic recovery, and the emergence of new economic powers, the number of trade communities increased to seven, accompanied by substantial structural reconfiguration. The previously unified China–US-centered community split, with each country forming the core of separate communities. The European Union and North American countries formed a highly integrated and clearly bounded bloc, while Russia became fully integrated into the China-centered trade system. This shift indicates a transition from major-power coordination to a bloc-based trade configuration. Consequently, the stability of the global trade network increasingly depends on internal cohesion within major trade blocs rather than on multilateral cooperation.

Overall, the evolution of the RTEAP-TN communities is not determined solely by geographic proximity. Instead, it reflects the coexistence of intensified regional clustering and the restructuring of cross-regional linkages. This finding is consistent with Tang et al. (2024), suggesting that regionalization trends are not confined to regional trade systems but are also evident in the global ready-to-eat Aquatic Products trade network [26].

### 3.2. Evolutionary Analysis of RTEAP-TN Sub-Trade Networks

The foregoing analysis of the community structure of the overall trade network in this study has revealed, at the macro level, the influence of geopolitical and other factors on trade patterns. On this basis, this section provides a detailed analysis of the sub-trade networks of six categories of ready-to-eat Aquatic Products from two perspectives: structural characteristics and core countries. By comparing the similarities and differences in the evolution of these sub-trade networks, the key sub-network categories that influence the evolution of the characteristics of the overall trade network are identified, and the differences in the roles of core countries across different sub-trade networks are further examined.

**Figure 7:**
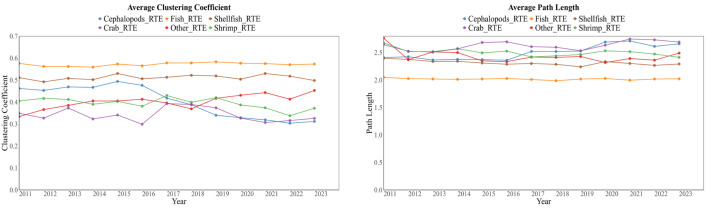
The temporal evolution of the average clustering coefficient and average path length of the six-category sub-trade network.

Subnetworks exhibit clear differences during their evolution, indicating that changes in the overall trade network cannot be simply regarded as the average result of the synchronous evolution of all product categories (Figure 7). Among them, the ready-to-eat fish trade network has the largest trade scale. The trends of its average clustering coefficient and average path length are highly consistent with those of the overall trade network. This indicates that the fish sub-network plays a dominant role in shaping the overall evolutionary characteristics of the RTEAP-TN. The ready-to-eat shrimp network also shows strong consistency with the overall network, but its influence ranks second. In contrast, the crab, shellfish, cephalopods, and other subnetworks exhibit more differentiated evolutionary paths. This indicates that the overall network dynamics are largely driven by categories with larger scale and denser connections, rather than representing the structural characteristics of all subcategories.

Although the six sub-trade networks differ in their evolutionary paths, they share certain common topological properties. The small-world index of all six subnetworks is greater than 1, indicating that they all exhibit small-world characteristics. Meanwhile, the cumulative degree distributions of all subnetworks show varying degrees of heavy-tailed behavior, suggesting that trade connections across different categories are concentrated in a few key countries. This implies that all RTEAP-TNs combine efficient connectivity with hub dependence to some extent. However, the degree of reliance on core nodes varies across categories. Some subnetworks have a relatively larger group of core countries and more alternative connections, whereas others rely more on a few key nodes to maintain overall connectivity. This difference provides a structural basis for the significantly different resilience observed across categories in subsequent vulnerability analysis (Supplementary S2).

Compared with previous studies that treat aquatic products as a single category, the classification-based comparison in this study reveals more refined structural differences. Existing studies have mainly discussed global Aquatic Products trade patterns based on the overall network [24,25,44]. In contrast, this study finds that the evolution of the overall trade network is not jointly determined by all subcategories, but is mainly driven by the fish sub-network, while other categories exhibit more differentiated structural paths. This suggests that conclusions based on aggregated networks may obscure important differences among subcategories.

**Figure 8:**
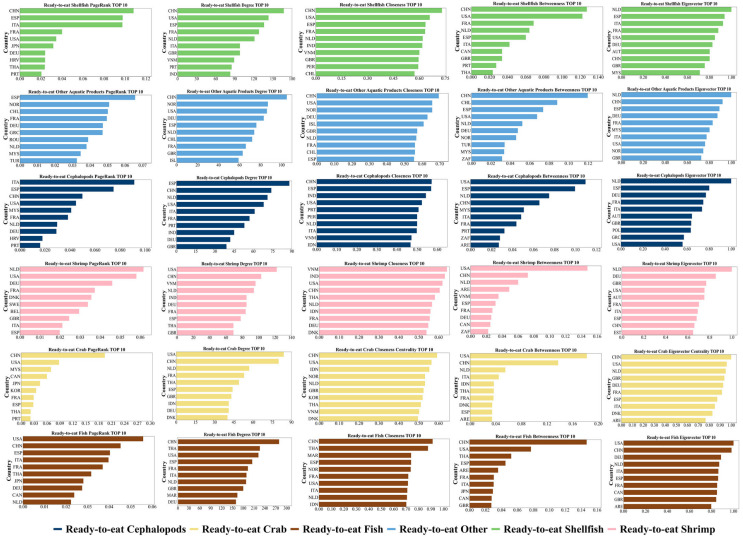
2023 Ranking of Major Trading Entities in Six Categories of ready-to-eat Aquatic Products trade networks Based on Centrality Metrics.

To further identify key trading entities across product categories, this study compares the centrality of major countries in the six subnetworks. Figure 8 shows that China and the United States occupy important positions in all six RTEAP-TNs, reflecting their broad influence in the global system. However, the composition of core countries varies across categories, showing clear category-specific differences. Specifically, Spain plays a prominent role in cephalopods and shellfish trade networks; Norway has stronger influence in other product categories; the Netherlands plays a significant role in the crab trade network; and Vietnam and Thailand show high importance in shrimp and fish trade, respectively. This indicates that the ready-to-eat Aquatic Products trade system is not a homogeneous market, but presents different combinations of core countries, connection patterns, and structural dependencies across categories. Analyses based solely on the overall trade network may overlook these category-level differences.

This finding is generally consistent with previous studies suggesting that a few core countries have long dominated global Aquatic Products trade (Gephart and Pace, 2015; Sun et al., 2025) [25,44]. However, this study further shows that different categories of ready-to-eat Aquatic Products do not share identical core country structures, but instead form category-specific configurations of core countries. This implies that the ready-to-eat Aquatic Products trade system exhibits stronger heterogeneity than the overall aquatic product trade network.

### 3.3. RTEAP-TN Vulnerability Analysis

To evaluate the structural stability of the RTEAP-TN, this section conducts a vulnerability analysis based on node attack simulation. In complex network structures, node attack refers to the process of altering the network topology by removing specific nodes and their associated edges, and is commonly used to simulate the impact of node failure on network function. According to different attack strategies, node attacks can be classified into two types: random attacks and targeted attacks. Random attacks refer to the random selection and removal of nodes with a certain probability, regardless of their importance in the network [45,46]. In the RTEAP-TN, such attacks can be analogous to disruptions caused by extreme climate events, natural disasters, or international trade fluctuations, leading to random node failures, local disconnections, and a gradual decline in overall network performance. In contrast, targeted attacks selectively remove key nodes in the network. This strategy directly disrupts the central backbone of the network and may lead to a rapid collapse of its overall structure. For example, under scenarios such as the COVID-19 pandemic, geopolitical conflicts, or regional environmental pollution, specific trade routes or core supply nodes may be directly disrupted, resulting in local paralysis or a sharp decline in the overall efficiency of regional or global trade networks.

**Figure 9:**
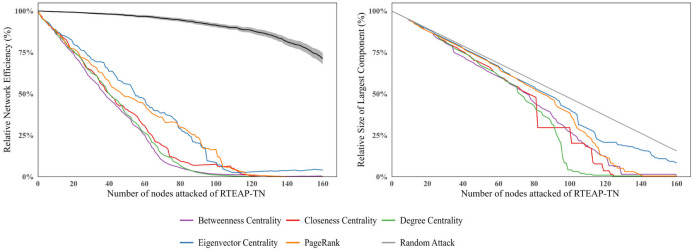
Changes in Overall Network Efficiency and Proportion of Largest Connected Component.

By simulating these two attack scenarios, the vulnerability of the RTEAP-TN was analyzed. The results show that the RTEAP-TN exhibits stronger stability under random attacks. As the proportion of removed nodes increases, the decline in network efficiency is more pronounced under targeted attacks. For example, when network efficiency decreases to 0.1, targeted attacks require the removal of only about 20% of nodes to achieve a level of disruption comparable to that under random attacks (Figure 9).

#### 3.3.1. Vulnerability Analysis of the Overall Trade Network

The simulation results indicate that trade power is highly concentrated in a few core countries, which constitute structural vulnerabilities of the RTEAP-TN. Under targeted attacks, the removal of approximately 50% of nodes leads to a decline in overall network efficiency to below 0.1. Among different attack strategies, betweenness centrality-based attacks are the most destructive, with both network efficiency and the proportion of the largest connected component showing abrupt declines. Degree centrality-based attacks are the second most destructive. This suggests that once a few core countries acting as “global trade bridges” are disrupted, the connectivity and efficiency of the entire trade network rapidly collapse, further confirming the critical role of core nodes in maintaining network stability.

#### 3.3.2. Vulnerability Analysis of the Sub-Trade Networks

**Figure 10:**
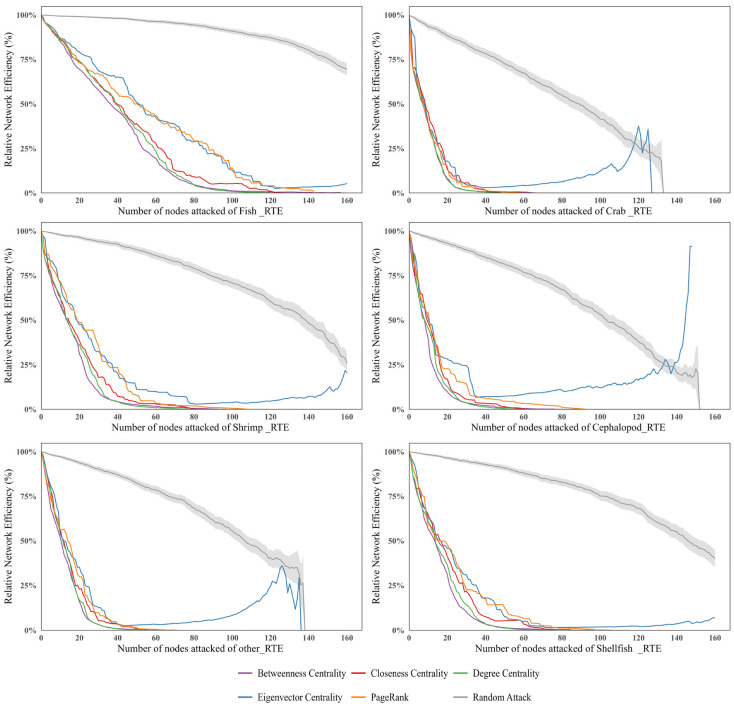
Changes in network efficiency.

The vulnerability simulation reveals heterogeneity across different categories of ready-to-eat Aquatic Products. The six subnetworks can be divided into two distinct groups: a robust fish network and a more vulnerable non-fish cluster. Specifically, the global ready-to-eat crab trade network exhibits the highest vulnerability (Figure 10). When approximately 16% of core nodes are removed, its network efficiency immediately drops below 0.1. As the proportion of removed nodes increases to about 22%, the network efficiency further declines to below 0.01, indicating that the network approaches collapse. Similarly, the ready-to-eat cephalopods, shrimp, shellfish, and other Aquatic Products trade networks experience a decline in network efficiency to below 0.1 after 20–30% of nodes are removed under targeted attacks. In contrast, the ready-to-eat fish trade network is significantly more stable. Even when more than 45% of nodes are removed under targeted attacks, its network efficiency remains above 0.1, demonstrating strong structural stability and risk resistance.

Within the sub-trade networks, attacks based on betweenness centrality are the most destructive, followed by those based on degree centrality. It should be noted that under adaptive attacks based on eigenvector centrality, as nodes are continuously removed, the remaining network may evolve into a directed acyclic graph or locally acyclic connected components in later stages. In such cases, the conventional spectral definition of eigenvector centrality no longer provides meaningful interpretation. Therefore, changes in eigenvector centrality are not considered in the vulnerability analysis.

**Figure 11:**
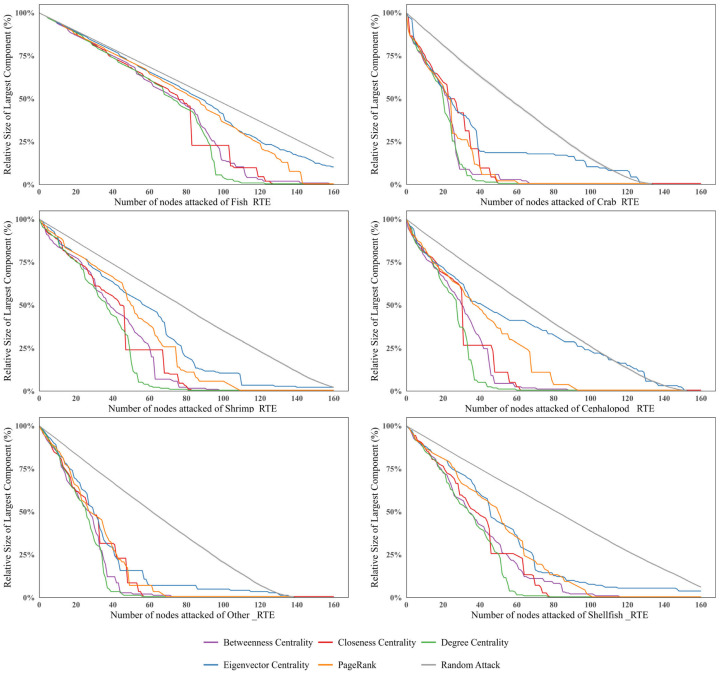
Changes in Network Efficiency and Proportion of Largest Connected Component in Sub-Trade Networks.

Further analysis of trade network characteristics shows that the non-fish RTEAP-TN exhibits a highly centralized structure, in which a few core countries dominate most trade flows. While this structure enhances local efficiency, it also makes the network more vulnerable under targeted attacks. In contrast, the ready-to-eat fish trade network has a larger group of core countries and denser inter-node connections. Such a structure effectively maintains network functionality under attacks, thereby providing stronger resistance to risks (Figure 11).

This finding further extends previous research on the vulnerability of global food trade networks. Karakoc and Konar (2021), Xu et al. (2024), and Ji et al. (2024) [12,13,40] all indicate that international trade networks are highly sensitive to the failure of key nodes, and that targeted attacks generally cause greater damage than random shocks. The results of this study are consistent with these findings, but further demonstrate that such vulnerability is not evenly distributed across different ready-to-eat Aquatic Products subnetworks. The fish network is relatively robust, whereas non-fish networks, especially the crab network, exhibit higher structural vulnerability.

Robustness tests show that, after changing the trade-record threshold, adjusting the edge-weight definition, and using the trade-value network instead of the trade-volume network, the results remain consistent with the main findings of this study. This indicates that the conclusions of this study are robust (Supplementary S3).

## 4. Discussion

This study finds that the global RTEAP-TN exhibits three key structural characteristics: high regional trade clustering, short interregional circulation paths, and a high concentration of core hub nodes. These structural features have dual effects on the trade network. On the one hand, the network enables cross-regional resource allocation with fewer intermediate links, thereby achieving higher circulation efficiency. On the other hand, the entire trade system accumulates non-negligible structural risks due to its heavy reliance on a small number of core countries and key circulation channels. This finding is consistent with previous studies on global food trade networks and Aquatic Products trade networks, which emphasize the coexistence of high connectivity and high concentration [8,24,44].

From the perspective of multiple concurrent crises, the vulnerability of the trade network is neither a result of routine trade fluctuations, nor an isolated shock caused by a single unexpected event, nor a simple accumulation of multiple risks. Instead, it arises from the interaction and mutual amplification of different types of risks. Climate-related risks directly disrupt the stability of raw material supply. Public health emergencies significantly reduce processing capacity and logistics efficiency. Geopolitical shifts reshape trade partnerships and market access rules. Meanwhile, the high concentration of the market further reinforces the dependence of the entire trade system on core nodes. When these risks simultaneously affect all stages of the value chain—including production, processing, transportation, industry governance, and final consumption—even minor disturbances in local markets can rapidly propagate along trade linkages and escalate into systemic risks across the entire chain.

Therefore, the dynamic changes observed in the trade network in this study fundamentally reflect the structural manifestation of the continuous accumulation and concentrated release of vulnerabilities in the global ready-to-eat Aquatic Products trade system under the normalization of multiple crises [3,4,6].

The analysis of core countries shows that the global RTEAP-TN is not supported by a group of functionally homogeneous countries, but rather by a set of key countries with distinct roles and clear division of functions. China, the United States, Thailand, France, and Spain have consistently occupied central positions in the network, but their roles differ significantly. China primarily serves as a comprehensive trade hub and network coordination node. The United States functions as a major global consumption market. Thailand acts as a prominent processing and re-export center. France and Spain demonstrate clear advantages in regional trade integration and specialized trade in specific product categories. These findings are highly consistent with previous studies identifying core countries in global Aquatic Products trade [25,28,44]. Building on this, the present study further clarifies that the value of core countries in the trade network lies not only in their trade scale and network centrality, but also in the specific functions they perform in linking supply and demand, organizing processing activities, and coordinating regional trade.

The analysis of trade community evolution indicates that the global ready-to-eat Aquatic Products trade pattern is shifting from a relatively dispersed and globally integrated model toward a dual structure characterized by deepening intra-regional trade clustering and the selective reconfiguration of interregional trade linkages. This finding is consistent with previous studies on the evolution of Aquatic Products trade networks under the continuous implementation of regional trade agreements and the restructuring of geopolitical landscapes [26,27].

This transformation reflects a fundamental shift in the underlying logic of trade organization. The core considerations of ready-to-eat Aquatic Products trade are no longer limited to cost efficiency and geographic proximity, but increasingly emphasize institutional compatibility, mutual recognition of inspection and quarantine standards, cold-chain logistics capacity, and supply chain security. The prolonged escalation of multiple crises has been a key driver of this shift. Public health emergencies have directly reduced the efficiency of global logistics integration. Geopolitical fragmentation has increased the uncertainty of interregional trade cooperation. Meanwhile, the increasing frequency of climate-related risks has intensified fluctuations in raw material supply and the likelihood of regional production adjustments.

Under these conditions, the evolution of the global RTEAP-TN has shifted from a traditional globalization model—focused on efficiency and broad global coverage of trade linkages—toward a structure that prioritizes supply chain security and resilience, with an emphasis on building more controllable and stable trade connections.

Further analysis of sub-trade networks reveals significant heterogeneity across product categories within the ready-to-eat Aquatic Products trade system. Although all six subcategories exhibit strong regional clustering and a high dependence on core hub nodes, they differ markedly in the composition of core participating countries, the degree of structural concentration, and their capacity to withstand external shocks. Among them, the fish sub-network shows the highest consistency with the overall trade network, confirming its central supporting role in the global system. In contrast, non-fish subnetworks, such as those for crab, shellfish, and cephalopods, exhibit a higher dependence on a limited number of core nodes and fewer alternative trade pathways. As a result, these networks are more vulnerable to external shocks and are more likely to experience disruption or breakdown.

This finding is consistent with previous research on agricultural trade networks, which emphasizes that different product categories exhibit differentiated structural resilience [20,23]. It also highlights a critical implication: the ready-to-eat Aquatic Products trade system should not be treated as a homogeneous market. Analyses based solely on aggregated trade data may obscure structural risks associated with high concentration in specific high-value subcategories.

The vulnerability simulation further confirms a key pattern: trade networks are significantly more sensitive to targeted disruptions of core nodes than to random failures of ordinary nodes. In particular, when shocks directly affect key nodes that serve as transit and bridging hubs, both network efficiency and the size of the largest connected component decline rapidly and substantially. This result clearly demonstrates that the stability of trade networks depends not on the number of ordinary nodes, but on whether core bridging nodes that connect interregional trade linkages can maintain stable operation. From the perspective of multiple crises, these core bridging nodes act as critical transmission hubs through which localized risks are amplified and propagated into systemic shocks across the entire trade system.

The findings of this study provide direct managerial implications for the seafood industry, particularly for enterprises in the ready-to-eat Aquatic Products sector.

First, in procurement strategy formulation, firms should not rely solely on price and short-term cost considerations when selecting supply sources. They should also evaluate the concentration of supply nodes within the global trade network, as well as their bridging functions and hub positions. For product categories that heavily depend on a limited number of core exporting or transit countries, priority should be given to diversifying sourcing strategies.

Second, in supplier selection and management, firms should formally incorporate “network position risk” into their supplier evaluation systems. Disruptions in suppliers located at key bridging nodes can have far greater impacts than those at ordinary nodes. Therefore, firms need to establish mechanisms for identifying critical suppliers, developing alternative sourcing options, and preparing contingency plans for unexpected disruptions.

Third, in end-to-end risk management, significant differences exist in the vulnerability of different product categories. Firms should adopt differentiated management strategies accordingly. For relatively robust categories, such as fish, efforts can focus on improving regional coordination efficiency and responsiveness to end-market demand. In contrast, for more vulnerable categories—such as crab, shellfish, and cephalopods—greater emphasis should be placed on strengthening safety stock buffers, developing alternative supply channels, and implementing dynamic risk monitoring for core nodes.

From the perspective of policymaking and industry governance, ensuring the security of global ready-to-eat Aquatic Products trade should not rely solely on the expansion of trade volume as the primary metric. Instead, greater attention should be given to the structural stability and resilience of the trade network. Governments and regulatory authorities should prioritize three key actions. First, they should strengthen dynamic monitoring of core nodes, high-risk product categories, and critical cross-border trade corridors, and establish efficient, full-chain risk early warning and emergency response systems. Second, they should continue to promote diversification of import sources and cultivate regional alternative trade nodes, thereby reducing excessive dependence on single core countries or key trade routes and mitigating structural concentration risks at the source. Third, they should deepen multilateral and regional economic cooperation, promote mutual recognition of food safety standards, coordinate inspection and quarantine procedures, and enhance the interconnectivity of cross-border cold-chain infrastructure, thereby reducing the negative impacts of institutional frictions and logistical bottlenecks on trade stability.

## 5. Conclusions

To fully understand the global ready-to-eat Aquatic Products trade system, it is crucial to conduct a systematic analysis of its trade network evolution and vulnerability. This study, based on global import and export data of ready-to-eat Aquatic Products from 2011 to 2023, employs complex network analysis to examine the evolution of its trade network. Utilizing Gephi 0.10.1 software, it provides a visualized presentation of the community characteristics of the network across different periods. Furthermore, by simulating targeted and random attacks, it systematically evaluated the vulnerability of the overall trade network and its six sub-trade networks. The main findings are as follows:

(1) The global RTEAP-TN exhibits typical small-world network characteristics, with a relatively high clustering coefficient and a short average path length. This indicates that the network maintains strong local connectivity while achieving efficient global connectivity. Meanwhile, the degree distribution of network nodes shows a clear heavy-tailed pattern and a hub-concentrated structure, suggesting that a small number of core countries control a large share of trade connections. While this structure enhances the efficiency of resource and information transmission, it also implies potential systemic risks, as disruptions to key nodes may lead to a rapid degradation of overall network functionality.
(2) Based on a comprehensive analysis of network indicators, including degree centrality, closeness centrality, and betweenness centrality, this study identifies China, the United States, Thailand, France, and Spain as core countries in the global RTEAP-TN, where they play critical roles.
(3) During the evolution process, the trade network gradually forms several communities with dense internal connections. The communities in Europe and North America exhibit clear boundaries, whereas those in regions such as Asia-Pacific and Africa show greater overlap and dynamism under the influence of geo-economic and geopolitical factors.
(4) In the sub-trade networks, all six categories of ready-to-eat Aquatic Products exhibit small-world characteristics. Their degree distributions also show varying degrees of power-law-like behavior in the upper tail and a pronounced hub-concentrated structure. The influence of countries varies across product categories. Spain plays a prominent role in cephalopods and shellfish trade, Norway dominates other product categories, the Netherlands holds a key position in crab trade, and Vietnam and Thailand serve as core actors in shrimp and fish trade, respectively.
(5) The vulnerability analysis further indicates that non-fish RTEAP-TNs are more sensitive to targeted attacks, with the crab trade network being the most vulnerable. The shrimp, shellfish, cephalopods, and other product networks exhibit similar levels of vulnerability, whereas the fish trade network shows stronger stability. Among different attack strategies, nodes with high betweenness centrality cause the greatest disruption, followed by those with high degree centrality and eigenvector centrality.

The policy and practical implications of this study lie in highlighting that the governance of the global ready-to-eat Aquatic Products supply chain should not focus solely on trade expansion, but should also emphasize network structural risks. For product categories that rely heavily on a limited number of core nodes, supply chain disruption risks can be mitigated by diversifying import sources, cultivating regional alternative nodes, strengthening monitoring and early warning systems for key nodes, and improving cross-national coordination mechanisms. For relatively robust trade networks, resilience can be further enhanced by improving regional cooperation and promoting mutual recognition of standards.

From an academic perspective, this study extends complex network analysis to the high-value and segmented field of ready-to-eat Aquatic Products trade. It not only reveals the evolutionary patterns of the global trade network at an aggregate level, but also compares six sub-trade networks in terms of structural characteristics, core country composition, and vulnerability. In doing so, it enriches the application of complex network theory in the study of international food trade and supply chain risk.

Nevertheless, several limitations should be acknowledged. First, in terms of data, the study relies on the United Nations Comtrade database, which may omit trade relationships from certain countries and informal trade flows, potentially leading to an underestimation of network density. Second, regarding timeliness, conclusions based on data from 2011–2023 may have limited explanatory power for future trade dynamics. Third, in terms of methodology, while complex network models are effective in analyzing macro-level topological structures, their ability to explain deeper driving mechanisms remains limited. Fourth, at the operational level, the selection of HS codes for ready-to-eat Aquatic Products may involve industry-specific biases, which could influence the analytical results.

Future research can be extended in three directions. First, combining complex network analysis with econometric models, shock-based models, or scenario simulations may help identify the driving forces of trade network evolution. Second, in-depth regional or country-level studies focusing on key product categories and critical nodes can enhance the specificity of policy recommendations. Third, integrating uncertainties such as climate change, geopolitical conflicts, and public health events into dynamic resilience assessments can provide more actionable evidence for ensuring the security of global ready-to-eat Aquatic Products supply chains.

## Figures and Tables

**Table 1:** Characteristic indicators of RTEAP-TN.

Year	Nodes	Edges	Degree of Connection	Connection Rate	Network Density	Average Path Length	Average Clustering Coefficient
2011	191	5863	0.000330066	30.69633508	0.161559658	1.993	0.599
2012	192	5915	0.000327203	30.80729167	0.161294721	1.993	0.590
2013	192	6040	0.000320653	31.45833333	0.164703316	1.98	0.590
2014	192	6020	0.000321683	31.35416667	0.164157941	1.974	0.593
2015	192	6116	0.000316797	31.85416667	0.166775742	1.951	0.606
2016	191	6200	0.000312694	32.46073298	0.170845963	1.970	0.596
2017	192	6129	0.000316147	31.92187500	0.167130236	1.962	0.603
2018	192	6180	0.000313621	32.18750000	0.168520942	1.946	0.601
2019	192	6247	0.000310364	32.53645833	0.170347949	1.970	0.610
2020	192	6123	0.000316447	31.89062500	0.166966623	1.994	0.610
2021	193	6158	0.000314653	31.90673575	0.166180915	1.962	0.612
2022	193	5973	0.000324075	30.94818653	0.161188472	1.982	0.607
2023	192	6026	0.000321319	31.22279793	0.162618739	1.975	0.604

**Table 2:** The development of modularization and changes in the number of communities.

Year	2011	2012	2013	2014	2015	2016	2017	2018	2019	2020	2021	2022	2023
Modularity	0.336	0.388	0.374	0.34	0.347	0.373	0.366	0.394	0.372	0.383	0.379	0.389	0.395
Number	6	6	7	6	5	6	4	6	6	5	6	6	7

## Data Availability

Publicly available datasets were analyzed in this study. The raw trade data for global bilateral trade in ready-to-eat Aquatic Products from 2011 to 2023 were obtained from the United Nations Comtrade Database. The data were processed through HS code screening, removal of trade records below 500 kg, network construction, and indicator calculation. The processed data supporting the findings of this study are available from the corresponding author upon reasonable request.

## Supplementary Materials

The following supporting information can be downloaded at: https://www.mdpi.com/article/10.3390/foods15101648/s1, Supplementary S1: HS Codes and Product Descriptions of Ready-to-Eat Aquatic Products; Supplementary S2: Small-world and scale-free indicators of the total trade network of ready-to-eat aquatic products and its six sub-trade networks from 2011 to 2023; Supplementary S3: Robustness test results.
