# Supplementary material for: Evolution and Vulnerability of the Global Ready-to-Eat Aquatic Products Trade Network: A Complex Network Analysis
Source: Foods. 2026 May 9;15(10):1648. doi: 10.3390/foods15101648 (PMC13205460; doi:10.3390/foods15101648)
Supplement: Supplementary file 1 [file foods-15-01648-s001.zip › foods-4258728-supplementary.pdf]

Supplementary S1

HS Codes and Product Descriptions of Ready-to-Eat Aquatic Products

HS Code	Product Description
030212	Fish: Pacific salmon (oncorhynchus nerka/gorbuscha/keta/tschawytscha/kisutch/masou/rhodurus), Atlantic salmon (salmo salar), Danube salmon (hucho hucho), fresh or chilled (excluding fillets, livers, roes and other fish meat of heading no. 0304)
030219	Fish: salmonidae, fresh or chilled, n.e.s. in item no. 0302.1 (excluding fillets, livers, roes and other fish meat of heading no. 0304)
030231	Fish: albacore or longfinned tunas (thunnus alalunga), fresh or chilled (excluding fillets, livers, roes and other fish meat of heading no. 0304)
030232	Fish: yellowfin tunas (thunnus albacares), fresh or chilled (excluding fillets, livers, roes and other fish meat of heading no. 0304)
030239	Fish: tuna, fresh or chilled, n.e.s. in item no. 0302.3 (excluding fillets, livers, roes and other fish meat of heading no. 0304)
030261	Fish: sardines (sardina pilchardus, sardinops spp.), sardinella (sardinella spp.), brisling or sprats (sprattus sprattus), fresh or chilled (excluding fillets, livers, roes and other fish meat of heading no. 0304)
030264	Fish: mackerel (scomber scombrus, scomber australasicus, scomber japonicus), fresh or chilled (excluding fillets, livers, roes and other fish meat of heading no. 0304)
030310	Fish: Pacific salmon, (oncorhynchus nerka/gorbuscha/keta/tschawytscha/kisutch/masou/rhodurus), frozen, (excluding fillets, livers, roes and other fish meat of heading no. 0304)
030322	Fish: Atlantic salmon (salmo salar) and Danube salmon (hucho hucho), frozen (excluding fillets, livers, roes and other fish meat of heading no. 0304)
030329	Fish: salmonidae, frozen, n.e.s. in item no. 0302.1 and 0302.2 (excluding fillets, livers, roes and other fish meat of heading no. 0304)
030341	Fish: albacore or longfinned tunas (thunnus alalunga), frozen (excluding fillets, livers, roes and other fish meat of heading no. 0304)
030342	Fish: yellowfin tunas (thunnus albacares), frozen (excluding fillets, livers, roes and other fish meat of heading no. 0304)
030349	Fish: tuna, frozen, n.e.s. in item no. 0303.4 (excluding fillets, livers, roes and other fish meat of heading no. 0304)
030371	Fish: sardines (sardina pilchardus, sardinops spp.), sardinella (sardinella spp.), brisling or sprats (sprattus sprattus), frozen (excluding fillets, livers, roes and other fish meat of heading no. 0304)
030374	Fish: mackerel (scomber scombrus, scomber australasicus, scomber japonicus), frozen (excluding fillets, livers, roes and other fish meat of heading no. 0304)
030510	Fish meal: fit for human consumption
030520	Fish: livers and roes, dried, smoked (whether or not cooked before or during the smoking process), salted or in brine
030530	Fish: fillets, dried, salted or in brine, but not smoked
030541	Fish: Pacific salmon (oncorhynchus nerka/gorbuscha/keta/tschawytscha/kisutch/masou/rhodurus), Atlantic salmon (salmo salar) and Danube salmon (hucho hucho), including fillets, smoked (whether or not cooked before or during the smoking process)
030542	Fish: herrings (clupea harengus, clupea pallasii), including fillets: smoked (whether or not cooked before or during the smoking process)
030549	Fish: smoked (whether or not cooked before or during the smoking process), n.e.s. in item no. 0305.4 (including fillets)
030551	Fish: cod (gadus morhua, gadus ogac, gadus macrocephalus), dried (whether or not salted but not smoked)
030559	Fish: dried (whether or not salted but not smoked), n.e.s. in item no. 0305.51
030561	Fish: herrings (clupea harengus, clupea pallasii), salted or in brine but not dried or smoked

HS Code	Product Description
030562	Fish: cod (gadus morhua, gadus ogac, gadus macrocephalus), salted or in brine but not dried or smoked
030563	Fish: anchovies (engraulis spp.), salted or in brine but not dried or smoked
030569	Fish: salted or in brine, but not dried or smoked, n.e.s. in item no. 0305.6
160411	Fish preparations: salmon, prepared or preserved, whole or in pieces (but not minced)
160412	Fish preparations: herrings, prepared or preserved, whole or in pieces (but not minced)
160413	Fish preparations: sardines, sardinella and brisling or sprats, prepared or preserved, whole or in pieces (but not minced)
160414	Fish preparations: tunas, skipjack and Atlantic bonito (sarda spp.), prepared or preserved, whole or in pieces (but not minced)
160415	Fish preparations: mackerel, prepared or preserved, whole or in pieces (but not minced)
160416	Fish preparations: anchovies, prepared or preserved, whole or in pieces (but not minced)
160419	Fish preparations: fish prepared or preserved, whole or in pieces (but not minced), n.e.s. in heading no. 1604
160420	Fish preparations: fish minced or in forms n.e.s. in heading no. 1604, prepared or preserved
160430	Fish preparations: caviar and caviar substitutes
030710	Molluscs: oysters, live, fresh, chilled, frozen, dried, salted or in brine (whether in shell or not)
030729	Molluscs: scallops (including queen scallops of the genera pecten, chlamys or placopecten), frozen, dried, salted or in brine (whether in shell or not)
030739	Molluscs: mussels (mytilus spp., perna spp.), frozen, dried, salted or in brine (whether in shell or not)
030760	Molluscs: snails (other than sea snails), live, fresh, chilled, frozen, dried, salted or in brine (whether in shell or not)
030799	Molluscs and other aquatic invertebrates: frozen, dried, salted or in brine (whether in shell or not), n.e.s. in heading no. 0307
150420	Fats and oils and their fractions: of fish, (excluding liver-oils)
150430	Fats and oils and their fractions: of marine mammals
160510	Crustacean preparations: crab, prepared or preserved
160520	Crustacean preparations: shrimps and prawns, prepared or preserved
160530	Crustacean preparations: lobster, prepared or preserved
030741	Molluscs: cuttle fish and squid, live, fresh or chilled (whether in shell or not)
030749	Molluscs: cuttle fish and squid, frozen, dried, salted or in brine (whether in shell or not)
030759	Molluscs: octopus (octopus spp.), frozen, dried, salted or in brine

Supplementary S2

Table S1: Scale-free measurement indicators of the ready-to-eat aquatic products trade network

year	alpha	Xmin	Fitted_range	Ks	Bootstrap_p	Lognormal_vs_powerlaw	Exponential_vs_powerlaw
2011	2.619556	58	[58, max]	0.121801	0	V=-2.5989, p=0.0094	V=-3.4700, p=0.0005
2012	2.593477	55	[55, max]	0.115269	0.004	V=-2.3651, p=0.0180	V=-2.9378, p=0.0033
2013	2.706865	60	[60, max]	0.107809	0.002	V=-2.1316, p=0.0330	V=-2.6121, p=0.0090
2014	2.361015	42	[42, max]	0.116473	0	V=-2.5454, p=0.0109	V=-2.6951, p=0.0070
2015	2.275056	38	[38, max]	0.110995	0	V=-2.8357, p=0.0046	V=-2.8534, p=0.0043
2016	2.356417	43	[43, max]	0.10372	0.002	V=-2.7199, p=0.0065	V=-2.9299, p=0.0034
2017	2.55649	52	[52, max]	0.107133	0	V=-2.1872, p=0.0287	V=-2.3504, p=0.0188
2018	2.551644	56	[56, max]	0.122202	0	V=-2.4421, p=0.0146	V=-3.1823, p=0.0015
2019	6.494219	173	[173, max]	0.125259	0.336	V=-0.6319, p=0.5275	V=-0.9199, p=0.3576
2020	6.120542	168	[168, max]	0.097282	0.828	V=-0.7594, p=0.4476	V=-1.4599, p=0.1443
2021	6.155151	168	[168, max]	0.098695	0.72	V=-0.8239, p=0.4100	V=-1.2075, p=0.2273
2022	2.6912	62	[62, max]	0.124326	0	V=-2.3957, p=0.0166	V=-3.2832, p=0.0010
2023	2.44843	48	[48, max]	0.122092	0.002	V=-2.5255, p=0.0116	V=-3.0338, p=0.0024

Table S2: Scale-free measurement indicators of the ready-to-eat shellfish trade network

year	alpha	Xmin	Fitted_range	Ks	Bootstrap_p	Lognormal_vs_powerlaw	Exponential_vs_powerlaw
2011	1.717644	5	[5, max]	0.109762	0	V=-3.1903, p=0.0014	V=-0.9730, p=0.3306
2012	4.000092	59	[59, max]	0.084798	0.818	V=-0.6146, p=0.5388	V=-0.6010, p=0.5478
2013	1.876939	8	[8, max]	0.087906	0.002	V=-2.2979, p=0.0216	V=-0.4173, p=0.6765
2014	1.89918	10	[10, max]	0.110944	0	V=-2.8025, p=0.0051	V=-1.8229, p=0.0683
2015	4.661239	75	[75, max]	0.100236	0.72	V=-0.3370, p=0.7361	V=-0.1998, p=0.8416
2016	4.238868	70	[70, max]	0.096166	0.664	V=-0.4510, p=0.6520	V=-0.2114, p=0.8326
2017	1.989963	12	[12, max]	0.098092	0.002	V=-2.3229, p=0.0202	V=-1.1326, p=0.2574
2018	2.110608	16	[16, max]	0.108124	0.002	V=-2.1473, p=0.0318	V=-1.4621, p=0.1437
2019	2.004692	13	[13, max]	0.107366	0.002	V=-2.5963, p=0.0094	V=-1.7008, p=0.0890
2020	4.400424	70	[70, max]	0.100334	0.716	V=-0.5986, p=0.5495	V=-0.5330, p=0.5941
2021	3.729422	58	[58, max]	0.07758	0.91	V=-0.6753, p=0.4995	V=-0.6288, p=0.5294
2022	3.430468	51	[51, max]	0.106455	0.308	V=-0.7387, p=0.4601	V=-0.6512, p=0.5149
2023	3.986467	61	[61, max]	0.08963	0.772	V=-0.4287, p=0.6681	V=-0.1412, p=0.8877

Table S3: Scale-free measurement indicators of the ready-to-eat cephalopod trade network

year	alpha	Xmin	Fitted_range	Ks	Bootstrap_p	Lognormal_vs_powerlaw	Exponential_vs_powerlaw
2011	2.135487	14	[14, max]	0.088003	0.052	V=-2.1372, p=0.0326	V=-1.2338, p=0.2173
2012	2.172093	14	[14, max]	0.083992	0.046	V=-1.7983, p=0.0721	V=-0.6601, p=0.5092
2013	2.109952	13	[13, max]	0.104778	0.002	V=-1.9908, p=0.0465	V=-0.9529, p=0.3406
2014	2.05658	12	[12, max]	0.108909	0	V=-2.2227, p=0.0262	V=-1.1780, p=0.2388
2015	2.358575	21	[21, max]	0.092996	0.064	V=-1.6909, p=0.0909	V=-1.3487, p=0.1774
2016	2.259512	17	[17, max]	0.090317	0.07	V=-1.7242, p=0.0847	V=-0.9100, p=0.3628
2017	2.328497	14	[14, max]	0.092985	0.066	V=-1.3639, p=0.1726	V=-0.1684, p=0.8663
2018	2.328085	12	[12, max]	0.086603	0.092	V=-1.3522, p=0.1763	V=0.0190, p=0.9849
2019	2.425817	12	[12, max]	0.075501	0.264	V=-1.0851, p=0.2779	V=0.4339, p=0.6643
2020	2.524161	16	[16, max]	0.096421	0.072	V=-1.4382, p=0.1504	V=-1.1584, p=0.2467
2021	2.525499	13	[13, max]	0.091292	0.05	V=-1.0256, p=0.3051	V=0.2401, p=0.8102

2022	2.368559	12	[12, max]	0.080706	0.216	V=-1.4338, p=0.1516	V=-0.6483, p=0.5168
2023	2.436271	12	[12, max]	0.093617	0.102	V=-1.3110, p=0.1899	V=-0.2423, p=0.8086

Table S4: Scale-free measurement indicators of the ready-to-eat fish trade network

year	alpha	Xmin	Fitted_range	Ks	Bootstrap_p	Lognormal_vs_powerlaw	Exponential_vs_powerlaw
2011	3.048379	70	[70, max]	0.100127	0.048	V=-1.5861, p=0.1127	V=-1.9237, p=0.0544
2012	3.205855	77	[77, max]	0.094948	0.11	V=-1.5319, p=0.1255	V=-1.8810, p=0.0600
2013	3.375413	87	[87, max]	0.104637	0.094	V=-1.4547, p=0.1458	V=-2.0198, p=0.0434
2014	6.61969	168	[168, max]	0.107834	0.534	V=-0.5167, p=0.6054	V=-0.8656, p=0.3867
2015	3.197951	80	[80, max]	0.105825	0.054	V=-1.6257, p=0.1040	V=-2.1743, p=0.0297
2016	2.305647	37	[37, max]	0.116694	0	V=-2.8994, p=0.0037	V=-3.0779, p=0.0021
2017	2.38269	41	[41, max]	0.115961	0	V=-2.7818, p=0.0054	V=-3.0003, p=0.0027
2018	2.385036	43	[43, max]	0.115612	0	V=-2.6854, p=0.0072	V=-3.1680, p=0.0015
2019	6.36326	156	[156, max]	0.096282	0.83	V=-0.4326, p=0.6653	V=-0.5458, p=0.5852
2020	3.162969	74	[74, max]	0.115754	0.01	V=-1.3833, p=0.1666	V=-1.6803, p=0.0929
2021	6.188089	146	[146, max]	0.095655	0.816	V=-0.3826, p=0.7020	V=-0.4262, p=0.6699
2022	2.931481	65	[65, max]	0.097568	0.052	V=-1.8737, p=0.0610	V=-2.3908, p=0.0168
2023	3.239686	78	[78, max]	0.10032	0.112	V=-1.6071, p=0.1080	V=-2.1677, p=0.0302

Table S5: Scale-free measurement indicators of the ready-to-eat shrimp trade network

year	alpha	Xmin	Fitted_range	Ks	Bootstrap_p	Lognormal_vs_powerlaw	Exponential_vs_powerlaw
2011	1.547747	2	[2, max]	0.124926	0	V=-3.7643, p=0.0002	V=-0.5321, p=0.5946
2012	2.136576	14	[14, max]	0.112128	0.008	V=-2.2321, p=0.0256	V=-2.3899, p=0.0169
2013	2.212312	14	[14, max]	0.119736	0.008	V=-1.6366, p=0.1017	V=-1.2623, p=0.2068
2014	1.739047	4	[4, max]	0.111034	0	V=-2.4814, p=0.0131	V=-0.1303, p=0.8964
2015	2.328633	19	[19, max]	0.135887	0	V=-1.9088, p=0.0563	V=-2.2006, p=0.0278
2016	1.636137	3	[3, max]	0.130348	0	V=-3.1237, p=0.0018	V=-0.7504, p=0.4530
2017	2.171578	14	[14, max]	0.114781	0.012	V=-2.1000, p=0.0357	V=-1.6123, p=0.1069
2018	2.18791	14	[14, max]	0.111951	0.006	V=-1.9758, p=0.0482	V=-1.3277, p=0.1843
2019	2.406363	20	[20, max]	0.103237	0.038	V=-1.7141, p=0.0865	V=-1.4996, p=0.1337
2020	2.288638	17	[17, max]	0.0894	0.106	V=-2.0170, p=0.0437	V=-1.9193, p=0.0549
2021	2.591297	23	[23, max]	0.109168	0.04	V=-1.3871, p=0.1654	V=-1.3801, p=0.1676
2022	2.415634	20	[20, max]	0.092765	0.124	V=-1.8085, p=0.0705	V=-1.8722, p=0.0612
2023	2.465038	22	[22, max]	0.103961	0.046	V=-1.8881, p=0.0590	V=-2.0963, p=0.0361

Table S6: Scale-free measurement indicators of the ready-to-eat crab trade network

year	alpha	Xmin	Fitted_range	Ks	Bootstrap_p	Lognormal_vs_powerlaw	Exponential_vs_powerlaw
2011	1.707619	2	[2, max]	0.093216	0.006	V=-1.7216, p=0.0851	V=1.6602, p=0.0969
2012	1.549511	1	[1, max]	0.102617	0	V=-2.8138, p=0.0049	V=2.3852, p=0.0171
2013	1.65916	2	[2, max]	0.08824	0.028	V=-2.4530, p=0.0142	V=0.7199, p=0.4716
2014	3.914582	27	[27, max]	0.101511	0.37	V=-0.2964, p=0.7669	V=0.2257, p=0.8215
2015	1.835128	4	[4, max]	0.115675	0.004	V=-1.9256, p=0.0542	V=-0.4679, p=0.6399
2016	4.925274	32	[32, max]	0.089581	0.834	V=-0.3040, p=0.7611	V=0.0194, p=0.9845
2017	1.635446	2	[2, max]	0.096754	0.004	V=-2.3596, p=0.0183	V=1.0405, p=0.2981
2018	4.982651	41	[41, max]	0.103225	0.702	V=-0.2202, p=0.8257	V=0.0605, p=0.9518
2019	1.71306	3	[3, max]	0.097096	0.004	V=-2.6625, p=0.0078	V=-0.0509, p=0.9594
2020	1.720155	3	[3, max]	0.089807	0.026	V=-2.2283, p=0.0259	V=-0.1414, p=0.8875

2021	4.448641	32	[32, max]	0.091327	0.682	V=-0.2476, p=0.8045	V=0.1335, p=0.8938
2022	5.074564	40	[40, max]	0.091344	0.856	V=-0.4642, p=0.6425	V=-0.5572, p=0.5774
2023	4.939727	35	[35, max]	0.080331	0.922	V=0.2094, p=0.8342	V=0.9044, p=0.3658

Table S7: Scale-free measurement indicators of the ready-to-eat other aquatic products trade network

year	alpha	Xmin	Fitted_range	Ks	Bootstrap_p	Lognormal_vs_powerlaw	Exponential_vs_powerlaw
2011	7.861894	55	[55, max]	0.119236	0.774	V=-0.4386, p=0.6610	V=-0.6841, p=0.4939
2012	7.286273	54	[54, max]	0.140289	0.38	V=-0.3240, p=0.7459	V=-0.4744, p=0.6352
2013	4.883974	48	[48, max]	0.145938	0.094	V=-0.6274, p=0.5304	V=-1.0110, p=0.3120
2014	1.841425	6	[6, max]	0.13091	0	V=-2.1916, p=0.0284	V=-1.2689, p=0.2045
2015	3.441476	33	[33, max]	0.103801	0.536	V=-1.1491, p=0.2505	V=-1.6490, p=0.0991
2016	7.351035	59	[59, max]	0.11157	0.868	V=0.0905, p=0.9279	V=0.3723, p=0.7096
2017	2.33493	15	[15, max]	0.110336	0.056	V=-1.6519, p=0.0986	V=-1.4747, p=0.1403
2018	2.403285	18	[18, max]	0.128208	0.004	V=-1.6210, p=0.1050	V=-1.7397, p=0.0819
2019	5.51052	58	[58, max]	0.125734	0.454	V=-0.6657, p=0.5056	V=-1.1211, p=0.2623
2020	7.252771	66	[66, max]	0.12445	0.654	V=-0.2813, p=0.7785	V=-0.2818, p=0.7781
2021	2.479428	20	[20, max]	0.128207	0.03	V=-1.7400, p=0.0819	V=-2.0258, p=0.0428
2022	2.804506	22	[22, max]	0.085529	0.386	V=-0.8220, p=0.4111	V=-0.2849, p=0.7757
2023	2.482447	19	[19, max]	0.114858	0.044	V=-1.2866, p=0.1982	V=-1.1278, p=0.2594

Table S8: Small-world indicators of the ready-to-eat aquatic products trade network

Year	Nodes	Edges	C_obs	L_obs	gamma	lambda	sigma	p_C
2011	191	4,379	0.51741	1.77966	1.061	1.0064	1.0543	0
2012	192	4,433	0.51896	1.77907	1.0629	1.0063	1.0563	0
2013	192	4,513	0.52222	1.78349	1.0622	1.0105	1.0511	0
2014	192	4,494	0.52041	1.78016	1.0661	1.0082	1.0574	0
2015	192	4,553	0.51888	1.77078	1.0603	1.007	1.053	0
2016	191	4,611	0.52808	1.77052	1.0599	1.007	1.0526	0
2017	192	4,542	0.52445	1.77372	1.0617	1.0056	1.0557	0
2018	192	4,613	0.52762	1.76991	1.0616	1.007	1.0543	0
2019	192	4,639	0.53115	1.76314	1.0583	1.005	1.053	0
2020	192	4,556	0.52591	1.76554	1.0607	1.0056	1.0548	0
2021	193	4,590	0.53075	1.77353	1.0611	1.0075	1.0532	0
2022	193	4,453	0.52969	1.78967	1.055	1.0083	1.0463	0
2023	192	4,499	0.52794	1.77209	1.0564	1.0044	1.0518	0

Table S9: Small-world indicators of the ready-to-eat fish trade network

Year	Nodes	Edges	C_obs	L_obs	gamma	lambda	sigma	p_C
2011	191	3966	0.48672	1.80716	1.0715	1.0069	1.0641	0
2012	192	4027	0.48904	1.80617	1.0725	1.0059	1.0662	0
2013	192	4128	0.49454	1.80825	1.0734	1.0101	1.0626	0

2014	192	4075	0.49014	1.81097	1.0752	1.0103	1.0642	0
2015	192	4105	0.48837	1.8029	1.0727	1.0077	1.0646	0
2016	191	4157	0.49882	1.80138	1.0707	1.0084	1.0618	0
2017	192	4130	0.49723	1.80443	1.0736	1.0088	1.0643	0
2018	192	4175	0.49884	1.80017	1.0729	1.0093	1.063	0
2019	192	4219	0.50389	1.7897	1.0695	1.0062	1.0629	0
2020	192	4128	0.49646	1.79232	1.0737	1.0067	1.0666	0
2021	193	4128	0.49701	1.80667	1.0713	1.0097	1.061	0
2022	193	3979	0.49532	1.82054	1.0629	1.009	1.0535	0
2023	192	4038	0.49684	1.80197	1.0682	1.0049	1.063	0

Table S10: Small-world indicators of the ready-to-eat crab trade network

Year	Nodes	Edges	C_obs	L_obs	gamma	lambda	sigma	p_C
2011	127	509	0.36229	2.52281	1.1329	1.0192	1.1115	0
2012	128	506	0.35908	2.48462	1.0843	1.0117	1.0718	0
2013	125	527	0.35544	2.4471	1.1	1.0155	1.0831	0
2014	127	505	0.34165	2.48281	1.1036	1.0099	1.0928	0
2015	116	513	0.35231	2.37841	1.0948	1.0017	1.0929	0
2016	118	509	0.3711	2.4653	1.1089	1.009	1.099	0
2017	134	638	0.39366	2.44799	1.1313	1.0241	1.1047	0
2018	134	655	0.37806	2.44002	1.136	1.0306	1.1023	0
2019	140	658	0.36505	2.48798	1.1451	1.0357	1.1057	0
2020	133	616	0.37435	2.48963	1.1545	1.0284	1.1226	0
2021	142	629	0.34589	2.57717	1.1966	1.0412	1.1492	0
2022	143	652	0.35261	2.61026	1.1736	1.0553	1.1122	0
2023	134	607	0.35141	2.49287	1.1514	1.0258	1.1225	0

Table S11: Small-world indicators of the ready-to-eat shrimp trade network

Year	Nodes	Edges	C_obs	L_obs	gamma	lambda	sigma	p_C
2011	160	1057	0.40048	2.32382	1.125	1.0253	1.0972	0
2012	157	1032	0.40246	2.33146	1.1477	1.0334	1.1106	0
2013	167	1064	0.39379	2.37378	1.1557	1.0363	1.1152	0
2014	164	1030	0.38474	2.34775	1.1511	1.0279	1.1198	0
2015	162	1062	0.39164	2.3193	1.1401	1.0255	1.1118	0
2016	166	1058	0.40066	2.3989	1.1528	1.0384	1.1101	0
2017	169	1225	0.39512	2.2917	1.1479	1.0204	1.1249	0
2018	167	1254	0.39703	2.30157	1.1666	1.0325	1.1299	0
2019	173	1275	0.38525	2.31489	1.1506	1.0325	1.1143	0
2020	172	1255	0.38815	2.3368	1.1485	1.0306	1.1143	0
2021	171	1286	0.39263	2.30224	1.1603	1.0246	1.1325	0
2022	169	1247	0.39741	2.32587	1.17	1.0304	1.1354	0
2023	170	1301	0.40054	2.27664	1.1845	1.0202	1.161	0

Table S12: Small-world indicators of the ready-to-eat shellfish trade network

Year	Nodes	Edges	C_obs	L_obs	gamma	lambda	sigma	p_C
2011	174	1401	0.3921	2.17547	1.0825	1.0157	1.0658	0

2012	181	1453	0.3844	2.19349	1.0657	1.0139	1.0512	0
2013	180	1519	0.38099	2.19013	1.0585	1.0297	1.0279	0
2014	179	1550	0.39588	2.174	1.0799	1.0201	1.0586	0
2015	175	1547	0.39943	2.14154	1.0645	1.0215	1.042	0
2016	182	1570	0.39739	2.16356	1.0747	1.0196	1.0541	0
2017	181	1657	0.39668	2.13781	1.0518	1.0174	1.0338	0
2018	179	1683	0.4032	2.14249	1.0678	1.0293	1.0374	0
2019	179	1682	0.39742	2.10043	1.0431	1.0099	1.0329	0
2020	176	1578	0.40427	2.12468	1.0595	1.0076	1.0515	0
2021	180	1672	0.40397	2.13135	1.0519	1.0139	1.0376	0
2022	173	1670	0.40625	2.09497	1.0559	1.0102	1.0452	0
2023	179	1663	0.40687	2.15084	1.0513	1.0184	1.0323	0

Table S13: Small-world indicators of the ready-to-eat cephalopod trade network

Year	Nodes	Edges	C_obs	L_obs	gamma	lambda	sigma	p_C
2011	167	1486	0.39135	2.14761	1.0676	1.0147	1.0521	0
2012	171	1500	0.39613	2.18163	1.0707	1.0167	1.0531	0
2013	172	1507	0.39092	2.15164	1.0823	1.0128	1.0687	0
2014	170	1466	0.38746	2.16422	1.0705	1.0191	1.0504	0
2015	172	1490	0.37857	2.12254	1.0615	1.0067	1.0545	0
2016	173	1516	0.38781	2.15849	1.0532	1.0183	1.0343	0
2017	169	1122	0.33329	2.29043	1.0843	1.0163	1.0669	0
2018	162	1007	0.30033	2.30734	1.0925	1.0172	1.074	0
2019	168	965	0.31393	2.41275	1.1466	1.0237	1.1201	0
2020	161	835	0.30653	2.47189	1.1727	1.0279	1.1409	0
2021	161	855	0.30605	2.45644	1.173	1.0291	1.1398	0
2022	154	768	0.29828	2.43995	1.1435	1.0102	1.1319	0
2023	153	772	0.30278	2.47566	1.1362	1.0256	1.1079	0

Table S14: Small-world indicators of the other ready-to-eat aquatic products trade network

Year	Nodes	Edges	C_obs	L_obs	gamma	lambda	sigma	p_C
2011	121	799	0.46489	2.38209	1.1918	1.0647	1.1194	0
2012	123	794	0.46213	2.37745	1.1582	1.0635	1.089	0
2013	130	805	0.43475	2.33679	1.1377	1.0395	1.0945	0
2014	127	826	0.42562	2.28196	1.1595	1.0357	1.1195	0
2015	124	824	0.42796	2.27039	1.0945	1.0269	1.0658	0
2016	133	890	0.42462	2.24197	1.101	1.0106	1.0895	0
2017	131	893	0.42019	2.26694	1.1268	1.0308	1.0931	0
2018	143	956	0.43013	2.35645	1.1226	1.0433	1.0761	0
2019	138	945	0.41576	2.26013	1.1024	1.0254	1.0751	0
2020	134	964	0.41485	2.18752	1.0873	1.0091	1.0775	0
2021	136	952	0.41532	2.22919	1.0915	1.0153	1.075	0
2022	130	924	0.42082	2.213	1.0988	1.0162	1.0812	0
2023	139	911	0.39077	2.25951	1.0766	1.0204	1.0551	0

Supplementary S3

The robustness test results show that, under different network construction methods, the core countries across different categories remain highly consistent overall. Major trading countries such as China, the United States, and Spain consistently occupy core positions in all models, indicating that the research results are highly robust. Only in a few categories do some peripheral countries, such as Thailand and France, show certain ranking fluctuations under different weighting settings, but these changes do not affect the overall judgment of the network structure.

The centrality rankings of various categories of ready-to-eat aquatic products in 2023 are shown in the table below. Here, **Threshold** indicates that the weight threshold for newly added edges in the ready-to-eat aquatic products trade network is set to 1,000; **Value-weighted** indicates that the edge weights in the newly constructed trade network are changed to trade value; and **Log-weighted** indicates that the edge weights in the newly constructed trade network are changed to log(1 + trade volume).

Metric: degree_total

Category: others

Rank	Threshold	Value-weighted	Log-weighted
1	China	China	China
2	Norway	United States	Norway
3	Germany	Germany	United States
4	Spain	Norway	Germany
5	United States	Netherlands	Spain

Category: cephalopods

Rank	Threshold	Value-weighted	Log-weighted
1	Spain	Spain	Spain
2	China	China	China
3	Netherlands	United States	Netherlands
4	United States	Netherlands	United States
5	Italy	Italy	Italy

Category: shrimp

Rank	Threshold	Value-weighted	Log-weighted
1	United States	United States	United States
2	Vietnam	China	China
3	China	France	Vietnam
4	Netherlands	Netherlands	Netherlands
5	India	Spain	India

Category: Crab

Rank	Threshold	Value-weighted	Log-weighted
1	United States	United States	United States
2	China	China	China
3	Netherlands	Netherlands	Netherlands

4	France	France	France
5	Thailand	Thailand	Thailand

Category: Shellfish

Rank	Threshold	Value-weighted	Log-weighted
1	China	China	China
2	United States	United States	United States
3	Spain	Spain	Spain
4	France	Netherlands	France
5	Netherlands	United Kingdom	Netherlands

Category: Fish

Rank	Threshold	Value-weighted	Log-weighted
1	China	China	China
2	Thailand	Thailand	Thailand
3	United States	United States	United States
4	Spain	Spain	Spain
5	Italy	France	France

Metric: betweenness

Category: Other

Rank	Threshold	Value-weighted	Log-weighted
1	China	China	China
2	Norway	Netherlands	United States
3	Germany	Czech Republic	Spain
4	Spain	United Kingdom	Chile
5	United States	United Arab Emirates	Netherlands

Category: cephalopods

Rank	Threshold	Value-weighted	Log-weighted
1	Spain	Netherlands	United States
2	China	Ireland	Netherlands
3	Netherlands	United States	Spain
4	United States	Germany	Malaysia
5	Italy	Portugal	Italy

Category: shrimp

Rank	Threshold	Value-weighted	Log-weighted
1	United States	Netherlands	United States
2	Vietnam	United States	China
3	China	France	United Arab Emirates
4	Netherlands	Switzerland	Netherlands
5	India	Germany	Spain

Category: Crab

Rank	Threshold	Value-weighted	Log-weighted
1	United States	United States	United States
2	China	China	China
3	Netherlands	Netherlands	Netherlands

4	France	Denmark	Italy
5	Thailand	Finland	Spain

Category: Shellfish

Rank	Threshold	Value-weighted	Log-weighted
1	China	Netherlands	United States
2	United States	Philippines	Netherlands
3	Spain	Luxembourg	China
4	France	France	France
5	Netherlands	United States	Canada

Category: Fish

Rank	Threshold	Value-weighted	Log-weighted
1	China	United Kingdom	China
2	Thailand	Turkey	Turkey
3	United States	Japan	United States
4	Spain	United Arab Emirates	United Arab Emirates
5	Italy	Luxembourg	France

Metric: closeness

Category: Other

Rank	Threshold	Value-weighted	Log-weighted
1	China	Norway	Norway
2	Norway	Turkey	China
3	Germany	Denmark	Chile
4	Spain	Chile	Spain
5	United States	Iceland	United States

Category: cephalopods

Rank	Threshold	Value-weighted	Log-weighted
1	Spain	United States	Spain
2	China	China	China
3	Netherlands	Spain	United States
4	United States	Vietnam	Italy
5	Italy	Thailand	India

Category: shrimp

Rank	Threshold	Value-weighted	Log-weighted
1	United States	China	Vietnam
2	Vietnam	United States	United States
3	China	Egypt	India
4	Netherlands	Canada	China
5	India	Netherlands	Thailand

Category: Crab

Rank	Threshold	Value-weighted	Log-weighted
1	United States	China	China

2	China	Russian Federation	United States
3	Netherlands	Canada	Indonesia
4	France	Myanmar	Thailand
5	Thailand	Indonesia	South Korea

Category: Shellfish

Rank	Threshold	Value-weighted	Log-weighted
1	China	China	China
2	United States	Peru	Spain
3	Spain	Spain	United States
4	France	Indonesia	France
5	Netherlands	Japan	Peru

Category: Fish

Rank	Threshold	Value-weighted	Log-weighted
1	China	China	China
2	Thailand	Thailand	Thailand
3	United States	Japan	Norway
4	Spain	United States	United States
5	Italy	Spain	Spain

Metric: eigenvector

Category: Other

Rank	Threshold	Value-weighted	Log-weighted
1	China	Turkey	Netherlands
2	Norway	Norway	Spain
3	Germany	Denmark	China
4	Spain	Greece	France
5	United States	United Kingdom	Germany

Category: cephalopods

Rank	Threshold	Value-weighted	Log-weighted
1	Spain	Italy	Netherlands
2	China	United States	Spain
3	Netherlands	South Korea	Italy
4	United States	China	France
5	Italy	France	Germany

Category: shrimp

Rank	Threshold	Value-weighted	Log-weighted
1	United States	United States	Netherlands
2	Vietnam	Uruguay	Germany
3	China	Mexico	United Kingdom
4	Netherlands	Italy	United States
5	India	Germany	France

Category: Crab

Rank	Threshold	Value-weighted	Log-weighted
1	United States	China	China

2	China	Malaysia	United States
3	Netherlands	Canada	France
4	France	United States	Netherlands
5	Thailand	Japan	United Kingdom

Category: Shellfish

Rank	Threshold	Value-weighted	Log-weighted
1	China	China	Spain
2	United States	Japan	Italy
3	Spain	South Korea	China
4	France	Thailand	France
5	Netherlands	Spain	Netherlands

Category: Fish

Rank	Threshold	Value-weighted	Log-weighted
1	China	United States	United States
2	Thailand	Japan	Spain
3	United States	Italy	China
4	Spain	France	Italy
5	Italy	Canada	Germany

The results show that, under different network construction methods, the vulnerability rankings of various ready-to-eat aquatic product trade networks remain generally stable. When network efficiency declines to 0.1, the ready-to-eat crab network requires the removal of only about 15% of nodes before network efficiency drops significantly. This indicates that its trade network is highly dependent on key country nodes and has relatively low network redundancy, making it the most vulnerable category among all product types. In contrast, the ready-to-eat fish network requires the removal of approximately 40% to 45% of nodes before network efficiency declines to 0.1, suggesting that this network has greater connectivity redundancy and more alternative paths, and therefore exhibits the strongest resistance to shocks (Figure 1)(Figure 2)(Figure 3)

The critical removal proportions for ready-to-eat shellfish, ready-to-eat other products, ready-to-eat cephalopods, and ready-to-eat shrimp are mostly concentrated around 18% to 20%, indicating that these categories have a moderate level of network resilience. Although some categories show slight variations under different weighting settings, such as ready-to-eat other products and ready-to-eat cephalopods fluctuating between 18% and 20%, and ready-to-eat shellfish being slightly higher than 20% in the Log-weighted model, the overall differences are small and do not alter the vulnerability assessment across product categories.

Overall, the vulnerability results obtained from the three network construction methods are highly consistent: the ready-to-eat crab network is the most vulnerable, the

ready-to-eat fish network is the most robust, and the remaining categories fall at an intermediate level. This suggests that the structural vulnerability of the ready-to-eat aquatic product trade network is not driven by a single weighting scheme, but is closely related to the trade structure of different product categories, the degree of dependence on core countries, and the number of alternative paths within the network. This result is consistent with the previous findings on core country identification and network structure analysis, further indicating that the conclusions of this study regarding the stability and vulnerability characteristics of the ready-to-eat aquatic product trade network are highly robust.

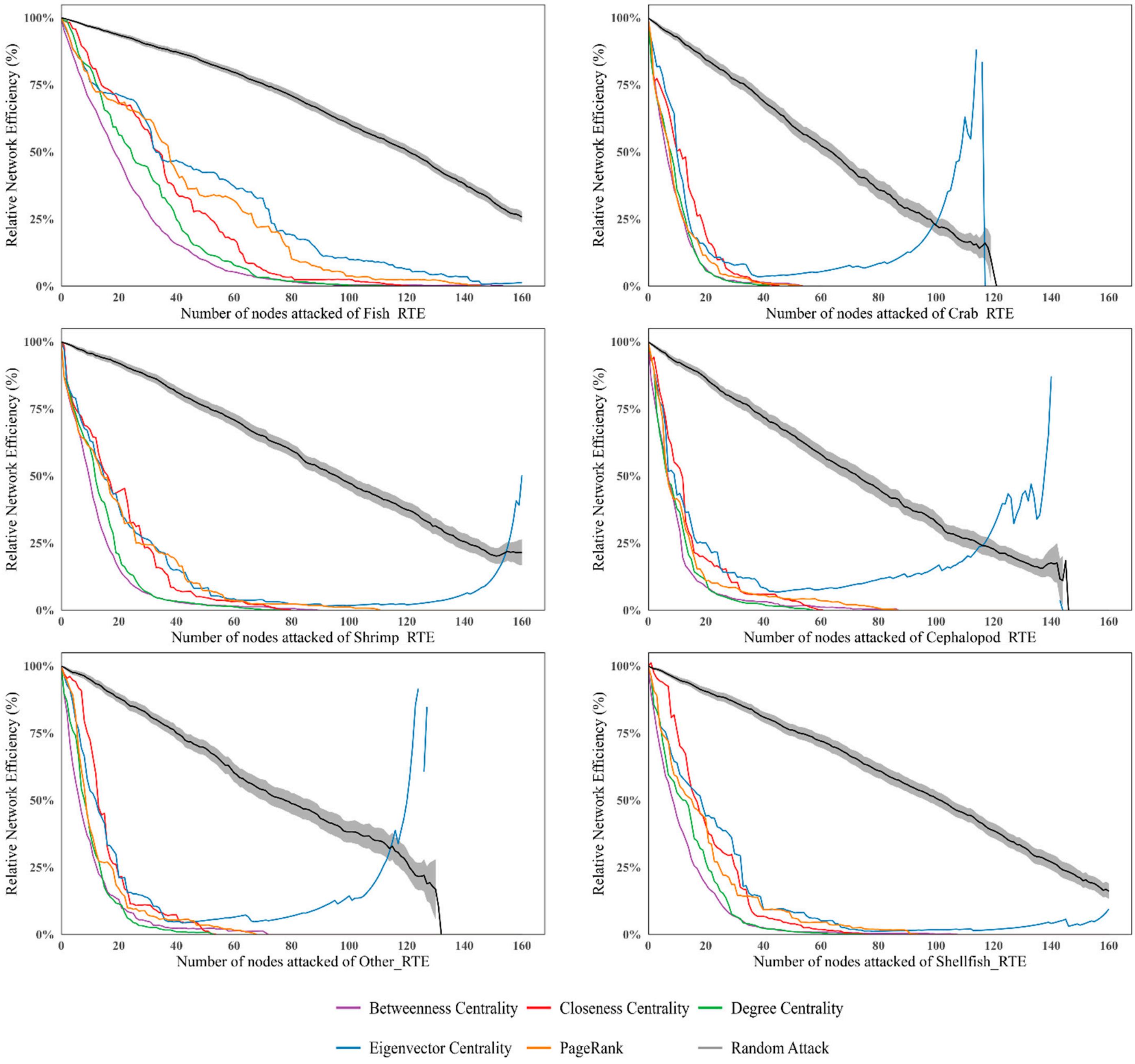

Figure S1: Network Efficiency Changes in the Ready-to-Eat Aquatic Products Trade Network under the 1,000 Threshold

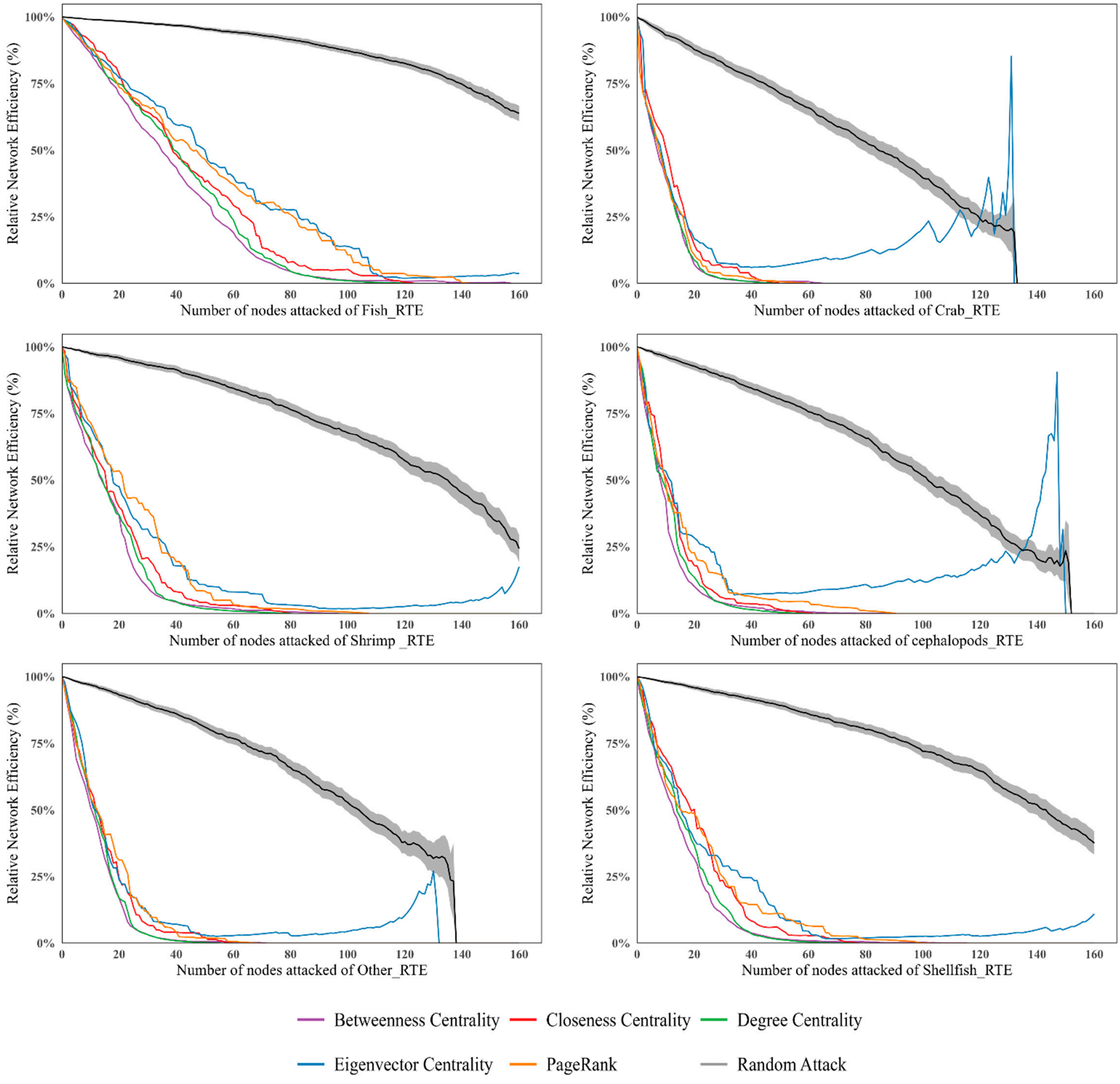

Figure S2: Changes in Network Efficiency of the Ready-to-Eat Aquatic Products Trade Network with Edge Weights Defined as log(1 + Trade Volume)

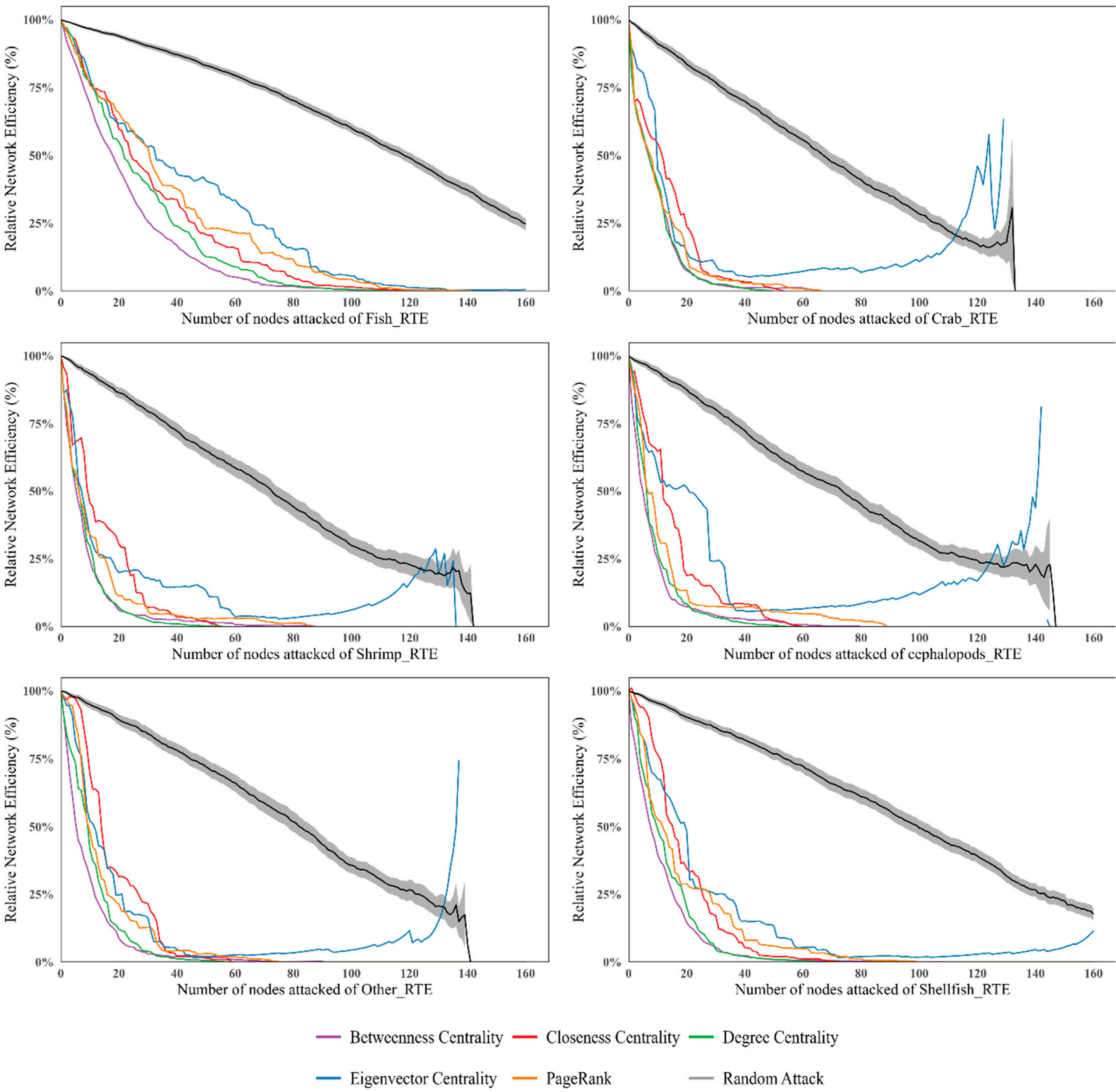

Figure S3: Network Efficiency Changes in the Ready-to-Eat Aquatic Products Trade Network under the 1,000 Threshold
